# Mitochondrial gene defects in Arabidopsis can broadly affect mitochondrial gene expression through copy number

**DOI:** 10.1093/plphys/kiad024

**Published:** 2023-01-27

**Authors:** Hiroki Ayabe, Atsushi Toyoda, Akitoshi Iwamoto, Nobuhiro Tsutsumi, Shin-ichi Arimura

**Affiliations:** Graduate School of Agriculture and Life Science, The University of Tokyo, 1-1-1 Yayoi Bunkyo-ku, Tokyo 113-8657, Japan; Department of Genomics and Evolutionary Biology, National Institute of Genetics, 1111 Yata, Mishima, Shizuoka, 411-8540, Japan; Faculty of Science, Kanagawa University, 2946 Tsuchiya, Hiratsuka, Kanagawa 259-1293, Japan; Graduate School of Agriculture and Life Science, The University of Tokyo, 1-1-1 Yayoi Bunkyo-ku, Tokyo 113-8657, Japan; Graduate School of Agriculture and Life Science, The University of Tokyo, 1-1-1 Yayoi Bunkyo-ku, Tokyo 113-8657, Japan

## Abstract

How mitochondria regulate the expression of their genes is poorly understood, partly because methods have not been developed for stably transforming mitochondrial genomes. In recent years, the disruption of mitochondrial genes has been achieved in several plant species using mitochondria-localized TALEN (mitoTALEN). In this study, we attempted to disrupt the *NADH dehydrogenase subunit7* (*NAD7*) gene, a subunit of respiratory chain complex I, in Arabidopsis (*Arabidopsis thaliana*) using the mitoTALEN method. In some of the transformants, disruption of *NAD7* was accompanied by severe growth inhibition and lethality, suggesting that *NAD7* has an essential function in Arabidopsis. In addition, the mitochondrial genome copy number and overall expression of genes encoding mitochondrial proteins were generally increased by *nad7* knockout. Similar increases were also observed in mutants with decreased *NAD7* transcripts and with dysfunctions of other mitochondrial respiratory complexes. In these mutants, the expression of nuclear genes involved in mitochondrial translation or protein transport was induced in sync with mitochondrial genes. Mitochondrial genome copy number was also partly regulated by the nuclear stress-responsive factors NAC domain containing protein 17 and Radical cell death 1. These findings suggest the existence of overall gene-expression control through mitochondrial genome copy number in Arabidopsis and that disruption of single mitochondrial genes can have additional broad consequences in both the nuclear and mitochondrial genomes.

## Teaser

Disruption of a single mitochondria-encoded gene causes widespread change in mitochondria and even nuclear gene expression, partially through increasing mitochondrial genome copy number.

## Introduction

Since the acquisition of mitochondria by eukaryotes, the mitochondrial genome has lost most of its genes and now contains only a few dozen genes encoding respiratory chain complexes and ribosome-related genes required for their expression (Unseld et al., 1997; Duchêne and Maréchal-Drouard, 2001). In angiosperms, mitochondria contain genes whose functions are poorly understood, partly because it is difficult to create deletion mutants (Larosa and Remacle, 2013; Chuah et al., 2015; Mileshina et al., 2015). Furthermore, there have been only a few successful cases of mitochondrial gene knockout (KO). Therefore, the functions of mitochondrial genes have been investigated indirectly using “surrogate mutants”, which are KO mutants of nuclear-encoded genes involved in post-transcriptional modifications of mitochondrial genes (Takenaka et al., 2019), and the functions of these genes remain elusive. In this study, we report the results of the disruption and phenotypic analysis of the *NADH dehydrogenase subunit7* (*NAD7*) gene using the mitoTALEN method in Arabidopsis (*Arabidopsis thaliana*). mitoTALEN is a genome editing tool that uses an artificial sequence-directed nuclease TALEN that localizes to mitochondria and that can directly disrupt genes on the mitochondrial genome. However, in angiosperms, mitoTALEN is only used to knock-out of non-essential genes until now, and the target genes in previous reports did not have a clear negative effect on plant survival. These target genes included cytoplasmic male sterility (CMS)-related genes (Kazama et al., 2019; Omukai et al., 2021; Kuwabara et al., 2022; Takatsuka et al., 2022), single KO of duplicated respiratory genes in *Arabidopsis thaliana* (Arimura et al., 2020), and point mutations on *nad9* (Forner et al., 2022). Moreover, disruptions by mitoTALEN sometimes cause large deletions (hundreds to thousands bps) and structural changes by illegitimate recombination around the targeted genes (Arimura et al., 2020), which may severely affect their function. Thus, it is unclear how the disruption of important genes affects mitochondrial or overall cellular function. To check this effect, we disrupted *NAD7* in Arabidopsis. *NAD7* gene is not only a candidate CMS-related gene in *Nicotiana sylvestris* (Chetrit et al., 1992; Pineau et al., 2005) but also an important gene as a subunit of respiratory complex I, we thought suitable for confirming the effect of disruption of important genes and selected as our target.

In our attempt to knock-out of *NAD7* in Arabidopsis, we found that *NAD7* KO caused lethality and could not investigate the its involvement to CMS. *nad7* KO also showed many other changes in the mitochondrial genome, and thus directed our attention to understanding the causes of these changes. *nad7* KO caused an increase in the mitochondrial genome copy number per cell and general induction of the expression of mitochondrial genes. Arabidopsis wild-type leaf cells normally contain 50–150 copies of the mitochondrial genome per cell (Preuten et al., 2010), and the copy number in the *nad7* KO was more than twice that of the wild-type. The mitochondrial genome copy number per cell has been shown to be positively associated with cell size in Arabidopsis (Preuten et al., 2010). In rice (*Oryza sativa*), more mitochondrial genomes are present in root tips and egg cells than in other tissues (Takanashi et al., 2006, 2010), suggesting that the copy number is somewhat regulated spatio-temporally. The mitochondrial copy number was found to be increased by inhibiting several processes, including organellar transcription (*Phage type RNA polymerase*; *RPOTmp*; Kühn et al., 2009), translation (*Ribosomal protein S10*; *RPS10*; Kwasniak et al., 2013), and repair and replication (*MutL protein homolog 1*; *MSH1* and *Recombination protein 3A*; *REC3A*; Shedge et al., 2010). However, the factors controlling mitochondrial genome replication are unclear, especially those factors that are related to external environmental changes (Gualberto et al., 2014; Morley et al., 2019). Studies of transcriptional and translational mutants have shown a relation between mitochondrial copy number and the expression of certain mitochondrial genes (Kühn et al., 2009; Kwasniak et al., 2013). However, these mutants showed general mitochondrial dysfunction, which made it difficult to see what triggered the increases. There are also several reports that some plants lacking such association (Woloszynska et al., 2006; Preuten et al., 2010). Thus, the conditions inducing increases in copy number and expression and the universality of their association remain unclear. Therefore, to investigate the response of the mitochondrial genome copy number and the expression level of mitochondrial genes in the presence of mitochondrial dysfunction, we further investigated this phenomenon using deletions of respiratory chain complex I subunits other than *NAD7*, deletions of other respiratory chain complexes, and inhibitor treatments.

## Results

### Trials to establish a mitochondrial genome knock-out of *nad7* in Arabidopsis

**Figure 1:**
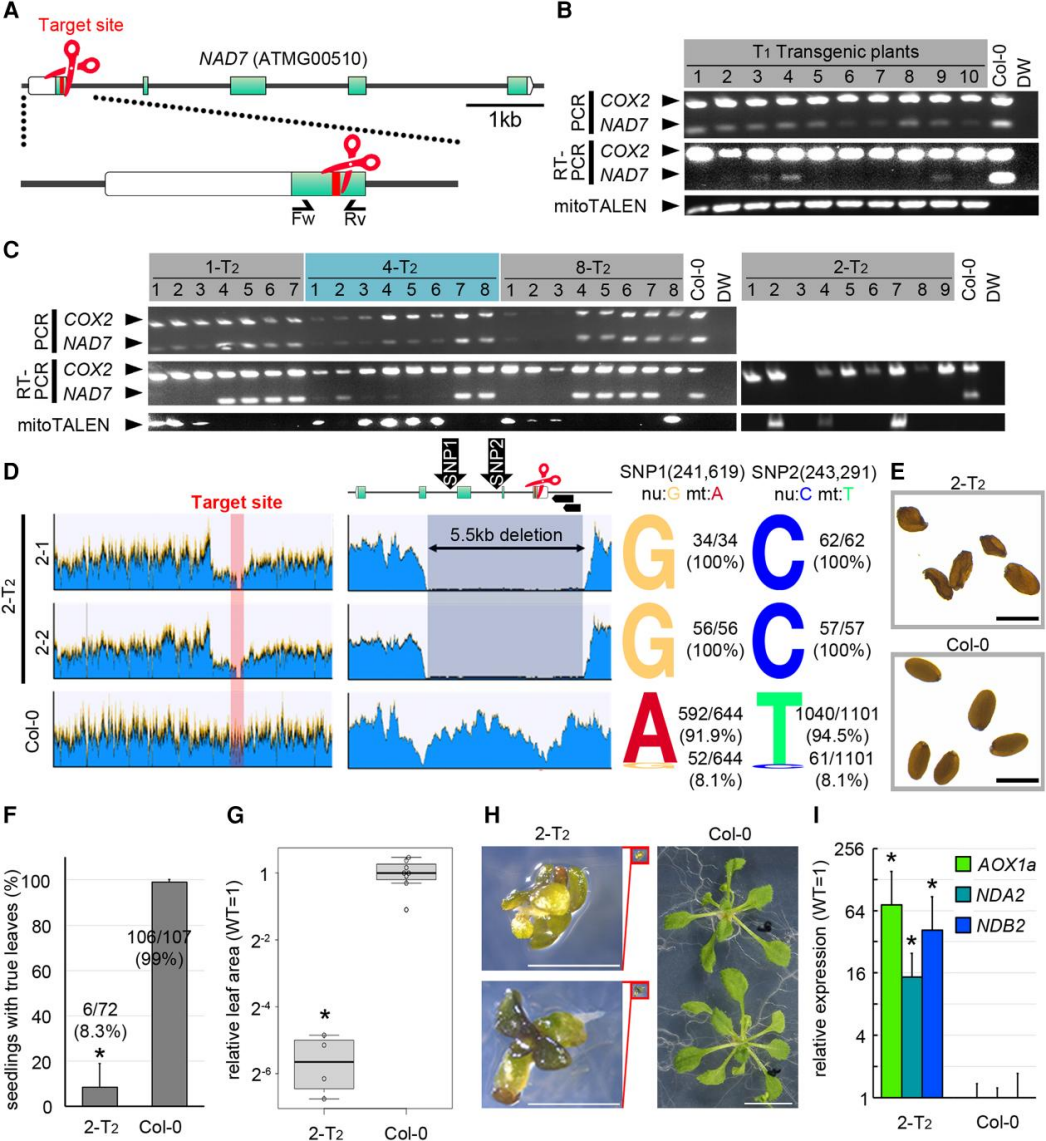
Genotypes and phenotypes of *nad7* KD or KO plants. A, The target and genomic structure of *NAD7*. Red indicates the target site of mitoTALEN. Boxes and green correspond to the five exons and coding sequences of *NAD7*. Arrows indicate the primer sets used in B–C. B, C, Genotyping of mitoTALEN-transfected T_1_ (B) and representative T_2_ lines (C). Multiplex PCR and RT-PCR using a mixture of two primer sets to amplify *NAD7* and *COX2* (control). The strain name of T_2_ corresponds to the T_1_ transformant in (B). D, Coverage graph of Illumina short reads from WGS mapped to the Arabidopsis wild-type mitochondrial genome (BK010421). From left to right, entire mitochondrial genome (Left), around the target (middle), and the ratio of the nuclear (NUMT) type reads to the mitochondrial type reads at two SNPs in the deletion region (right). The top row of the middle column shows the target sequence and its surrounding region, and the two black arrowheads on the right side show the ORFs (ORF107C and ORF141). E–H, Phenotypic analysis in 2-T_2_. Seed morphology (E). Survival rates of seedlings (F), values are mean ± SD of three independent growth trials on 1% sucrose × 1/2 MS solid medium. 2-T_2_ (*n* = 72), wild type (*n* = 108). Leaf area (G) and its representative phenotype (H) at 3 weeks after vernalization of individuals grown to true leaf expansion. 2-T_2_: *n* = 4, wild type: *n* = 8. In boxplot, horizontal lines are medians, boxes reach from the first to the third quartile. Whiskers mark the minimal (lower whisker) and maximal (upper whisker) data points within 1.5 times the interquartile range from the first and third quartile, respectively. Circles are outliers beyond the whiskers. Expression of mitochondrial dysfunction responsive genes (I). Each gene was standardized by *ACT2*, and relative values (wild type as 1) are shown. Values are means ± SD of 2-T_2_: *n* = 8, wild-type: *n* = 6. * indicates statistical significance by Student's *t* test (*P* < 0.05). Scale bars = 1 mm in (E), 2 mm in (H).

To introduce the CMS phenotype into Arabidopsis, we tried to knock out the mitochondrial gene *NAD7* by mitoTALENs. The target sites of the mitoTALENs, where double-strand breaks (DSBs) are made, were located within the first exon of *NAD7*. The mitoTALEN expression vectors were introduced into the nuclei of Arabidopsis Col-0 by the floral dipping method. Because previous studies showed that mitoTALENs caused large deletions (hundreds to thousands of bps) in the mitochondrial genomes, we carried out PCR of the *NAD7* target site to check the copy number of targets by comparing the intensity of PCR bands. In most of the T_1_ transformants, decreased amounts of *NAD7* PCR products were observed, confirming that at least *nad7* knock-down (KD) individuals were successfully obtained. However, even in plants with the severest reduction in *NAD7* band intensity, weak *NAD7* bands were still detected. They may have come from false-positive PCR amplifications of nuclear mitochondrial sequences (NUMTs) that resemble the mitochondrial gene. To distinguish these *NAD7* sequences in the nuclear and mitochondrial genomes, we performed RT-PCR. Almost all *NAD7* transcripts were expected to be derived from the mitochondrial genome, because the promoter sequence and RNA-polymerase types used were mitochondria-specific (Kühn et al., 2009; Garcia and Sanchez-Puerta, 2021), and actually the expression of NUMT genes were usually very low (Klepikova et al., 2016). No *NAD7* transcript was detected by RT-PCR in some of the T_1_ transformants (Figure 1B). So, it is expected that these plants have either no *NAD7* gene in their mitochondrial genome or a substantially reduced number of mitochondrial genomes with *NAD7*.

To check the deletion sites more precisely, we performed the same genotyping analysis on their self-pollinated T_2_ generation. In most of the strains (29 out of 30 strains analyzed), the copy number of *NAD7* was reduced when the mitoTALEN expression cassette was present in the nuclear genome, but when the cassette was lost from the nuclear genome by Mendelian segregation, the copy number of mitochondrial *NAD7* was restored to the same level as the wild-type (Figure 1C: 1-T_2_, 4-T_2_, 8-T_2_). This may be because the gene disruption in the parental T_1_ strain was incomplete, and the few remaining intact copies of the mitochondrial genome were replicated again by escaping from the mitoTALEN cleavage. On the other hand, only one strain, the T_2_ generation of strain No. 2 (2-T_2_), did not show any *NAD7* transcripts regardless of the presence or absence of the mitoTALEN expression cassette (Figure 1C: 2-T_2_). Thus, this line appears to have been derived from the parental line, some of whose cells were complete *nad7* KO (knock-out). To investigate the deleted region in 2-T_2_, the predicted NAD7 KO, WGS analysis was performed on two individuals (Figure 1D and Supplemental Figure 1). In both cases, the read coverage was clearly reduced in a 5.5 kb region containing the target (averaging about 3% of the read level in the wild-type Figure 1D: center). To determine whether the reads mapped in this 5.5 kb region were derived from the remaining non-disrupted wild-type mitochondrial genome or from a NUMT, two single-nucleotide polymorphisms (SNPs) between NUMT and mitochondrial *NAD7* sequences were utilized to check their origin (NUMT *NAD7*: ON220560.1; Fields et al., 2022, mitochondrial *NAD7*: BK010421; Sloan et al., 2018. The SNPs positions shown in Figure 1A are corresponded to BK010421). In the wild-type, more than 90% of the SNPs were mitochondrial (and therefore the remaining were NUMT-derived), whereas in 2-T_2_, 100% of the two SNPs were nuclear (NUMT)-type sequences, and no mitochondrial type was detected. This suggests that there is no intact wild-type mitochondrial genome in 2-T_2_ (Figure 1D: right). In addition to *NAD7*, two ORFs (ORF107C and ORF141) with unknown functions were identified in the deleted region. The remaining ends next to the deleted region were recombined into a distinct region by homologous recombination (Supplemental Figure 1). And these structural changes didn’t result in the appearance of any new chimeric open reading frames. For the other regions, no substantial reduction in coverage was observed compared with the wild-type, and no deletion mutations were found in important genes other than *NAD7*. These results indicate that *nad7* was likely knocked out in 2-T_2_.

Unlike the wild-type, 2-T_2_ produced wrinkled seeds (Figure 1E). In the early growth, only 8.3% of the sown seeds produced a seedling with true leaves on solid MS medium containing 1% sucrose (Figure 1F). Even among those that survived after leaf development, a significant delay in growth was observed at 3 weeks after vernalization, as shown by leaf areas that were less than 5% of those of the wild-type at the same age (Figure 1, G and H). Eventually all individuals died before bolting, so it was not possible to adequately examine the fertility of the floral organs for the involvement of *NAD7* in CMS trait. To see if *NAD7* disruption affected the expression of the mitochondrial dysfunction-responsive genes, we analyzed the expressions of several mitochondrial genes involved in mitochondrial stress response (*Alternative oxidase 1a*; *AOX1a*, *Alternative NAD(P)H dehydrogenase A2*; *NDA2*, and *Alternative NAD(P)H dehydrogenase B2; NDB2*; Clifton et al., 2006) by RT-qPCR. Each of these genes was strongly induced in 2-T_2_ (Figure 1I). These results suggest that deletion of the mitochondrial *NAD7* gene in Arabidopsis is nearly lethal, while its deletion in *Nicotiana sylvestris* had a relatively minor effect.

### Mitochondrial genome copy number per cell is increased in *nad7* KO 2-T_2_

**Figure 2:**
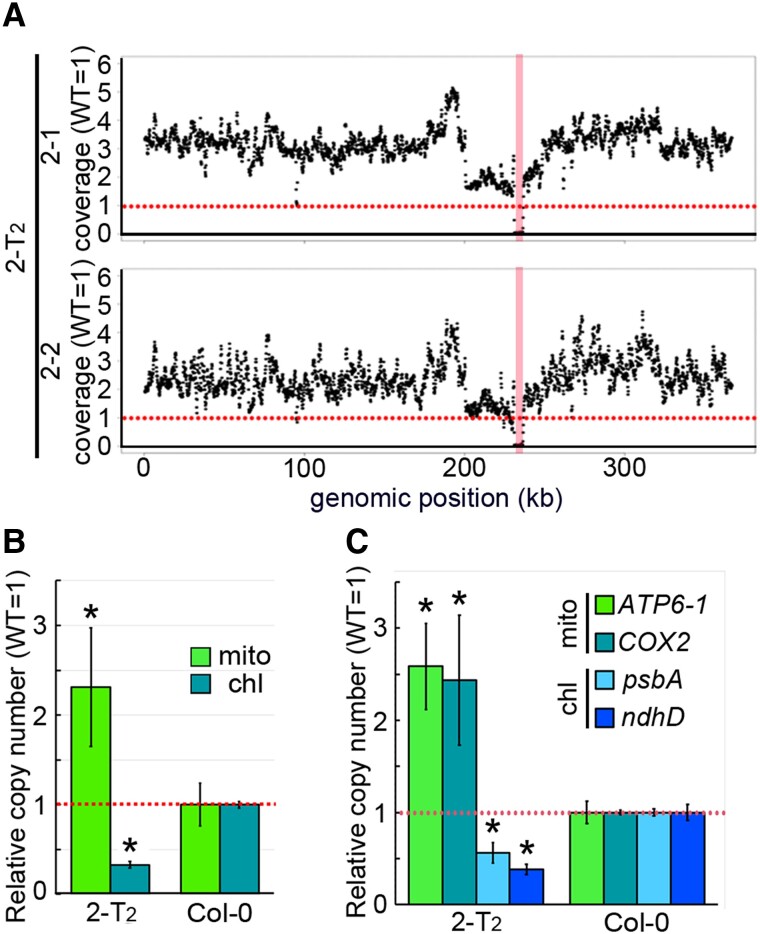
Mitochondrial genome relative coverage in *nad7* KO. A, Relative coverage variation of mitochondrial genome. B, Organelle genome copy number per cell estimated by WGS. Relative values (wild-type as 1) are shown. The vertical red bands indicate the 5.5 kb deleted region in (A). C, Organelle genome copy number per cell estimated by qPCR. Each gene was standardized by *TUA6*, and relative values (wild-type as 1) are shown. Horizontal red dotted line means the average wild type's copy number for each genomic region (A). For (B) and (C), values are means ± SD of 2-T_2_: *n* = 2, wild-type: *n* = 4. * indicates statistical significance by Student's *t* test (*P* < 0.05). chl, chloroplast genome encoded genes; mt, mitochondrial genome encoded genes.

From the WGS data obtained from 2-T_2_ individuals, we noticed that the number of reads mapped on the mitochondrial genome was higher than that of the wild-type throughout the mitochondrial genome (Figure 2A; except for the deletion region around the target). The mitochondrial genome copy number per cell, estimated from the total number of reads mapped to the nuclear and mitochondrial genomes, was approximately 2.3-fold higher in 2-T_2_ than in the wild-type (Figure 2B; 2–1: 2.78-fold, 2–2: 1.85-fold). This trend was also supported by the copy number estimated by qPCR (Figure 2C). In contrast, chloroplast genome copy number per cell in 2-T_2_ was significantly decreased. This phenomenon was not observed in other mutants with increased mitochondrial genome copy numbers (see following sections), and would be indirectly caused by its strong growth delay. These results suggest that the mitochondrial genome copy number per cell is increased in *nad7* KO 2-T_2_ compared with the wild-type.

### The increase of mitochondrial genome copy number is caused by a decrease of *NAD7* transcripts

**Figure 3:**
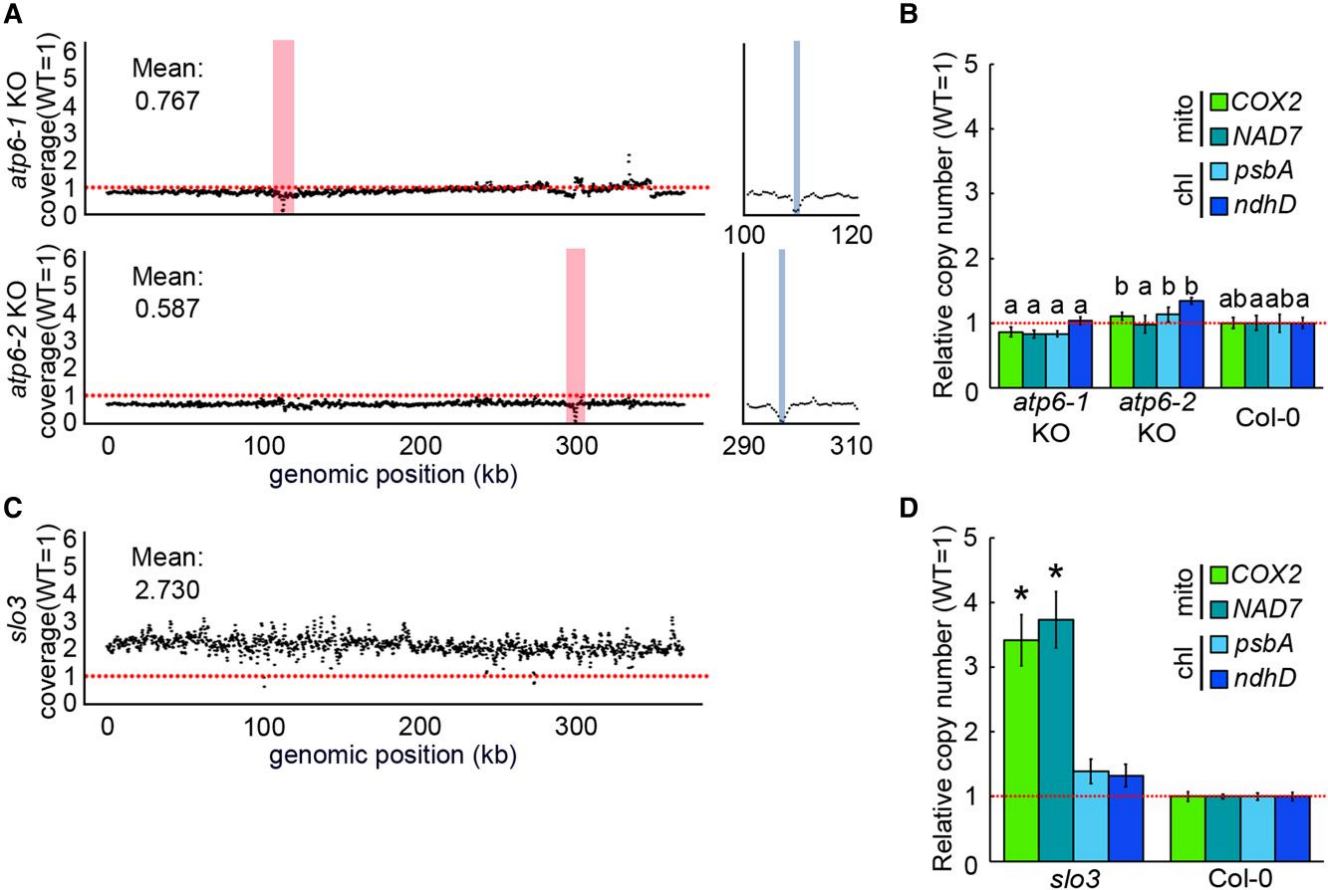
Depletion of functional *NAD7* mRNA causes mitochondrial genome copy number increase. A, C, Relative coverage variation of mitochondrial genome in *atp6-1* and *atp6-2* KO (A), and *slo3* (C). Red vertical bar indicates the target site of mitoTALEN. The right column is a magnified area around the target site, and the gray indicates the deleted regions in each strain, and horizontal red dotted line indicate means the wild type's copy number for each genomic region. B, D, Organelle genome copy number per cell estimated by qPCR for *atp6-1* and *atp6-2* KO (B), and *slo3* (D). Organelle genome copy number per cell estimated by qPCR. Each gene was standardized by *TUA6*, and relative values (wild-type as 1) are shown. Values are means ± SD of *n* = 3 or more for each strain. The different letters indicate statistical significance between groups by a one-way ANOVA with post hoc Tukey's test (*P* < 0.05) in (B), and * indicates statistical significance by Student's *t* test (*P* < 0.05) in (D).

2-T_2_ had an increased number of mitochondrial genomes (Figure 2). There are two possible causes for this increase, 1) deletion itself of the mitochondrial genome by mitoTALEN, and 2) mitochondrial dysfunction through deletion or lack of *NAD7* transcripts and/or the downstream product. To test the possibility No. 1, we checked the copy number of mitochondrial DNA in the other mitochondrial genome-edited plants in which non-essential genes were knocked out. The WGS results (NCBI accession; DRA010153) of plants with edited mitochondrial genome by mitoTALEN (*atp6-1* and *atp6-2* KO; both of which show the normal growth phenotype like wild-type) were analyzed to calculate organelle genome copy numbers. Both mutants showed a slight decrease in the overall mitochondrial genome copy number per cell compared with the wild-type, with several hundred bp deletions around each target (Figure 3A). qPCR analyses of *atp6-1* and *atp6-2* also showed no significant increase in mitochondrial genome copy number (Figure 3B). These results suggest that deletion of the mitochondrial genome itself is not the cause of copy number increase. To check another possibility, we performed an NGS analysis of the mutant *slow growth 3* (*slo3*) to examine the effect of reduced *NAD7* transcript levels. The *SLO3* gene is a nuclear-encoded PPR (pentatrico peptide repeat) protein that localizes to mitochondria and is required for the splicing of the *NAD7* intron 2. So, the KO mutant *slo3* is an indirect *nad7* KD mutant with reduced *NAD7* functional transcript levels that are less than a few percent of those in the wild-type (Hsieh et al., 2015). The coverage of the entire mitochondrial genome by the Illumina short reads of *slo3* was 2–3 times higher than the coverage in the wild-type (Figure 3C). This higher coverage in *slo3* was confirmed by qPCR (Figure 3D). These results suggest that the increase in mitochondrial genome copy number per cell observed in 2-T_2_ is not solely induced by the mitochondrial genome deletion by mitoTALEN, but by a shortage of *NAD7* transcript or its resultant mitochondrial dysfunction.

### Two *nad7* mutants showed increased accumulation of transcripts of many other mitochondrial genes

**Figure 4:**
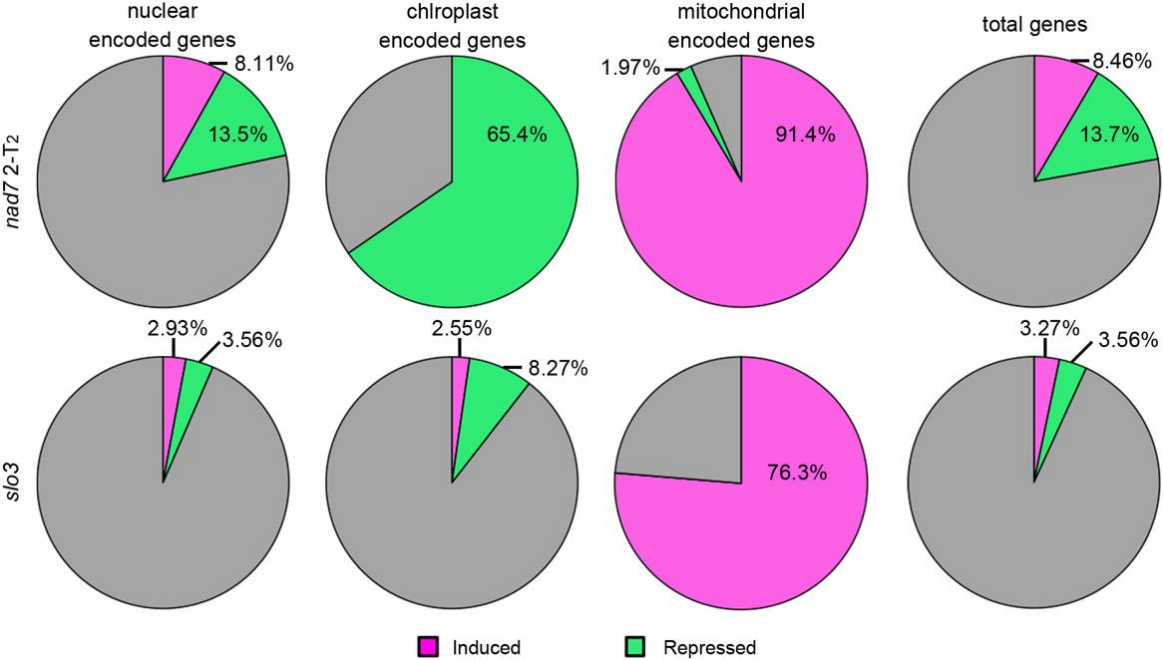
Transcriptional profiles in *nad7* KO and *slo3*. The proportion of differentially expressed genes (DEGs) against total genes encoded in each genome. From left to right, DEGs proportion among nuclear-encoded genes, mitochondrial-encoded genes, plastid-encoded genes, and the total genes (total genes encoded in nuclear, mitochondrial, and chloroplast genomes). DEGs with log2 FC (fold change) > 1 are shown as induced genes (magenta), and genes with log2 FC < −1 are shown as suppressed genes (green).

RNA-seq analyses of genes encoded in the nuclear, mitochondrial and plastid genomes were performed in 2-T_2_ and *slo3* and compared with the wild-type. For the differentially expressed genes (DEGs) detected in the comparison between wild-type and the mutants, the proportions of DEGs against total genes encoded in each genome were calculated (Figure 4, Supplemental Table 1–3). A significant higher proportion of induced DEGs were observed for mitochondrial genome (91.4%; 2-T_2_% and 76.3%; *slo3*) compared with the nuclear genome (8.11%; 2-T_2_% and 2.93%; *slo3*) (*P*< 0.05; fisher exact test). Moreover, the opposite trend was observed for the repressed DEGs, where their proportions in the mitochondrial genome (1.97%; 2-T_2%_ and 0%; *slo3*) were significantly lower than those in the nuclear genome (13.5%; 2-T_2_% and 3.56%; *slo3*) (*P* < 0.05. fisher exact test). These results suggest that the mutants that showed increased mitochondrial genome copy number per cell also showed overall induction of the expression of mitochondrial genes (i.e. genes encoded in mitochondrial genome).

### Increased mitochondrial genome copy number is observed in a wide range of mitochondrial respiratory failure besides *NAD7*

**Figure 5:**
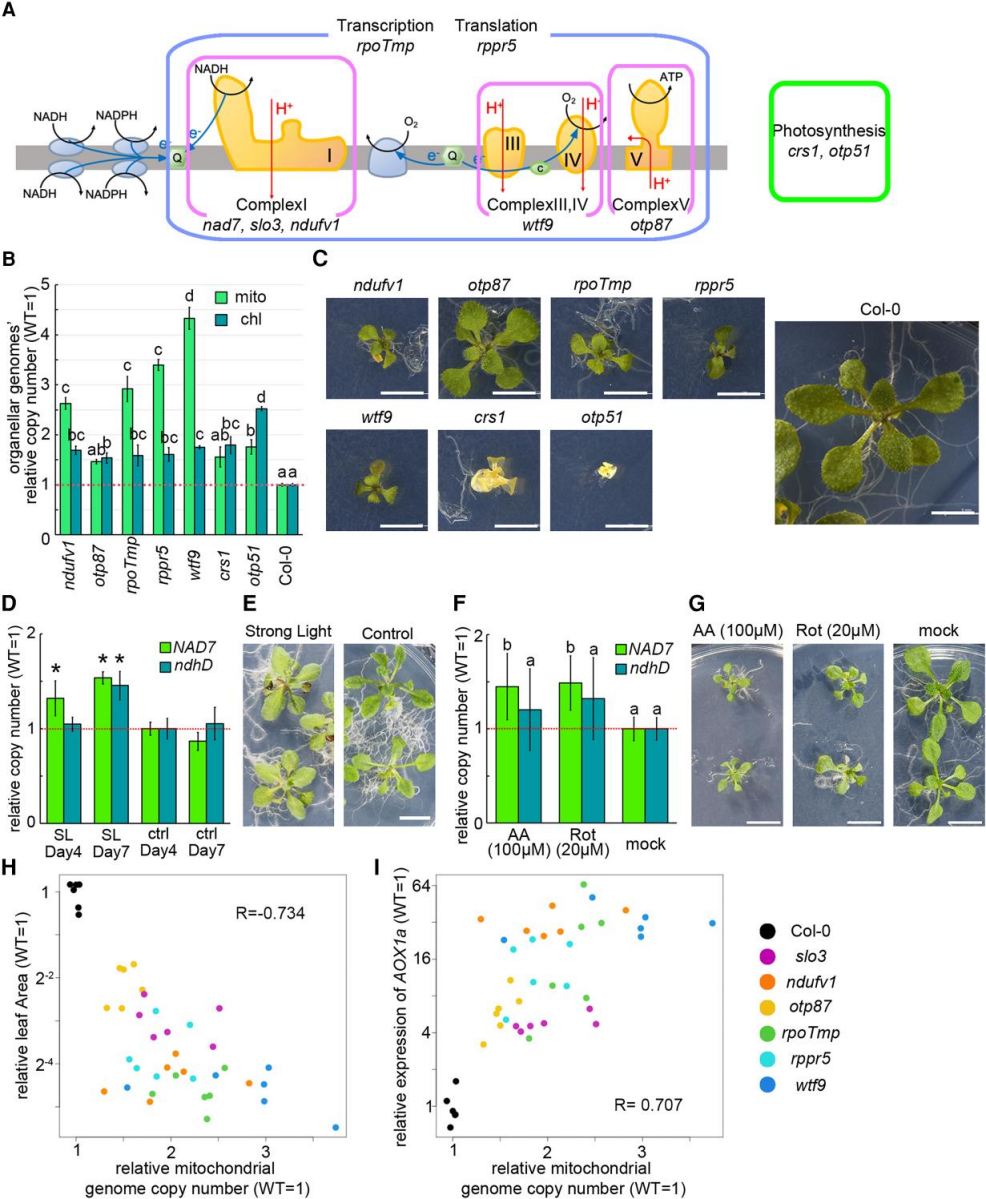
Genome copy numbers in various mitochondrial dysfunction mutants. A, Schematic drawing of the mitochondrial respiratory chain, showing which function is affected in each mutant. B, Organelle genome copy number per cell estimated by WGS. Relative values (wild-type as 1) are shown. Values are means ± SD of three samples per strain (one sample consists of 5–20 individuals in bulk; only *rppr5* has two samples). C, Phenotypes of each mutant at 3 weeks after vernalization. All photos are at equal magnification. D–G: High light treated wild-type organelle genome copy number (D) and its phenotype (E). The plants were grown for 4 and 7 days under high light (750 µmol/m^2^sec) or control conditions (80 µmol/m^2^sec). Respiratory inhibitor-treated wild-type organelle genome copy number (F) and its phenotype (G); plants were grown for 14 days on AA (100 µM), Rotenone (20 µM), or inhibitor-free (mock) solid medium. Each gene was standardized by *TUA6,* and relative values (wild-type as 1) are shown. Values are means ± SD of *n* = 4 (D) and *n* = 8 (F). The different letters indicate statistical significance between groups by a one-way ANOVA with post hoc Tukey's test (*P* < 0.05) in B and F, and * indicates statistical significance by Student's *t* test (*P* < 0.05) in D. H, I, Scatter plots of the comparison of copy number and the expression of *AOX1a* (H) and leaf area (I). R in the figure is Pearson's product rate correlation coefficient. *n* = 6 for each strain. Scale bar = 5 mm in (C), and 1 cm in (E, G).

We observed a mitochondrial genome copy number increase in 2-T_2_ and *slo3*, which are mutants with reduced *NAD7* transcript levels. To examine if this increase is also observed for mitochondrial dysfunctions caused by disruptions of other genes, five nuclear genome-encoding T-DNA insertion mutants with dysfunctions directly or indirectly on the respiratory chain (*51 kDa subunit of complex I*; *ndufv1*, *organellar transcript processing 87*; *otp87*, *rpoTmp*, *Ribosomal pentatricopeptide repeat protein 5*; *rppr5*, and *What this factor 9*; *wtf9*) and two chloroplast mutants with dysfunctions in photosystem II (*Chloroplast splicing factor 1*; *crs1* and *organellar transcript processing 51; otp51*) were analyzed. The mutations of the mutants are shown in Figure 5A and Supplemental Table 4, and their phenotypes at 3 weeks after vernalization are shown in Figure 5C. Four of the 5 mitochondrial dysfunctional mutants (all except for *otp87*, which showed the mildest growth retardation) showed significantly increased mitochondrial genome copy number per cell compared with the wild-type (Figure 5B and Supplemental Figure 2). The chloroplast genome copy number per cell was also significantly higher in all seven lines than in the wild-type, although the increase was not as large as that of the mitochondrial genome. These results indicate that a wide range of mitochondrial dysfunction cause an increase in mitochondrial genome copy number. Therefore, we hypothesized that the increase is induced by mitochondrial dysfunction rather than a lack of specific transcripts in the mutants. To confirm whether the increase can be induced without a shortage of any transcript, wild-type plants were treated with respiratory inhibitors [Rotenone and Antimycin A (AA)] or high light conditions (750 µmol/m^2^sec), where mitochondrial function is thought to be required for photorespiration and oxidation of excess reduced cofactors (Noguchi and Yoshida, 2008). Mitochondrial genome copy number was significantly higher in the treated plants than in the untreated control with both high light (Figure 5, D and E) and respiratory inhibitors (Figure 5, F and G). These results support the hypothesis that genome copy number increase is a response to stresses that require the enhancement or restoration of mitochondrial function. To examine the relationship between stress intensity and copy number increase, we compared this relationship in 6 mitochondrial mutant lines (*slo3*, *ndufv1*, *otp87*, *rpoTmp*, *rppr5*, and *wtf9*) and the wild-type. To examine the magnitude of growth retardation, we used leaf area. To examine the magnitude of the stress response, we used the expression of *AOX1a*, a stress marker gene whose transcription is induced under mitochondrial dysfunction (Clifton et al. 2006). Mitochondrial genome copy number was negatively and positively correlated with leaf area (Figure 5H) and *AOX1a* expression (Figure 5I), respectively. These results suggest that the increase in mitochondrial genome copy number is induced by mitochondrial dysfunction at multiple sites other than *NAD7*, and that the degree of increase is positively associated with stress caused by mitochondrial dysfunction. Thus, there appears to be a link between the mitochondrial stress and mitochondrial genome copy number.

### Mitochondrial genome copy number increase per cell in mutants cannot be explained by endoreduplication

In the previous sections, we observed the mitochondrial genome copy number increase in various mutants. However, the results were calculated by dividing the number of mitochondrial genomes by the number of nuclear genomes. Therefore, the mitochondrial genome copy number increase could be due to a reduced nuclear ploidy. To test this possibility, we measured the nuclear ploidy of *slo3*, *ndufv1*, and *rpoTmp* by flow cytometry. Mean nuclear ploidy level is given by the C-value, where 1C equals the DNA content of monoploid, unreplicated nuclei in haplophasic tissue. The C-value of each strain was either comparable with or significantly higher than that of the wild-type (Supplemental Figure 3A; Mean C-value *slo3*: 15.8, *ndufv1*: 16.1, *rpoTmp*: 13.4, Col-0: 13.9). The mitochondrial genome copy number per nuclear copy number in these samples confirmed a significant increase in two of the three strains (Supplemental Figure 3B). These results indicate that the increase in the mitochondrial genome copy number per cell could not be explained by lower ploidy, so it must have been due to an actual increase in the mitochondrial genome copy number in each cell.

### Global expression of mitochondrial genes is commonly induced in mutants with increased genome copy number

**Figure 6:**
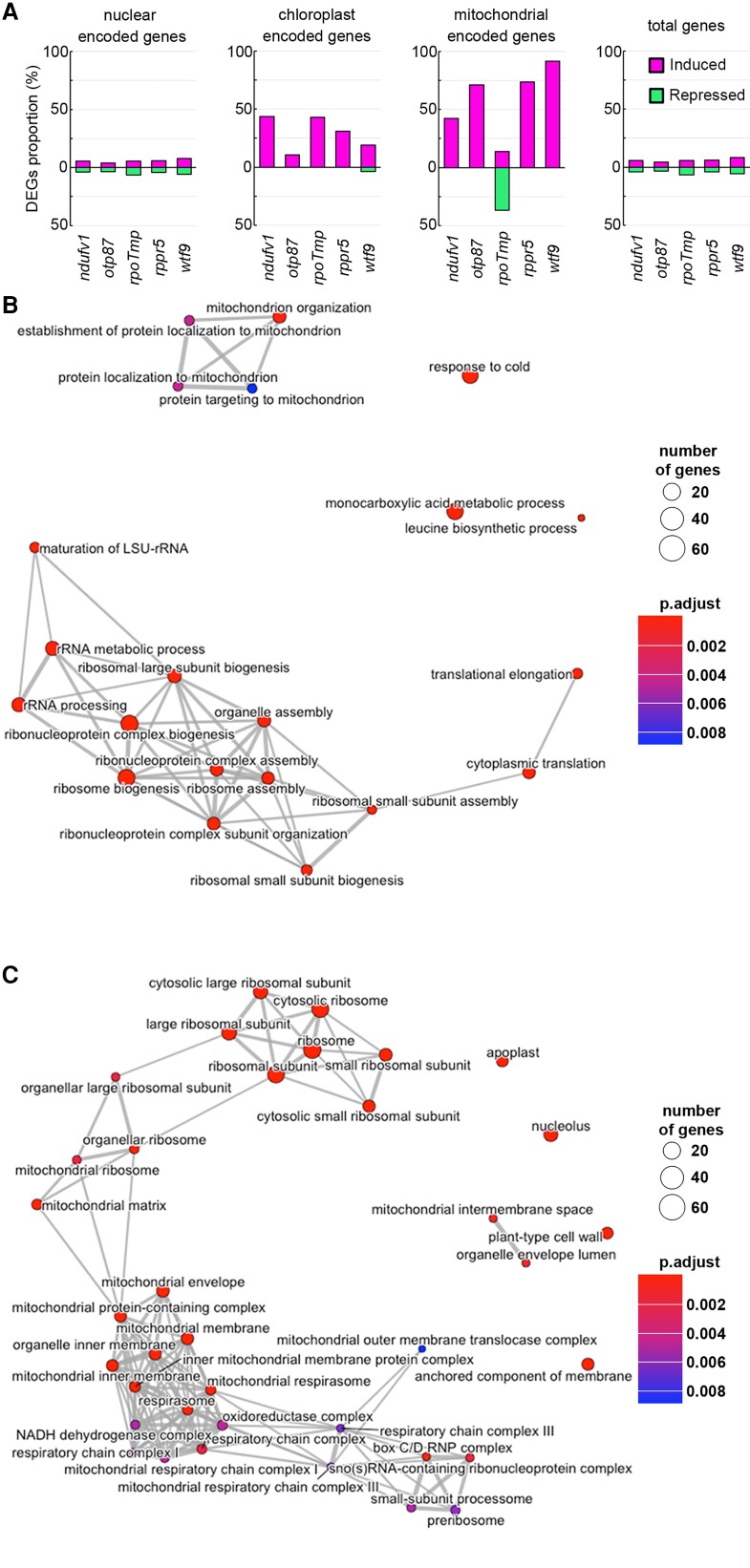
Transcriptional profiles in mutants with various mitochondrial dysfunctions. A, The proportion of differentially expressed genes (DEGs) against total genes encoded in each genome. From left to right, DEGs proportion among nuclear-encoded genes, mitochondrial-encoded genes, plastid-encoded genes, and the total genes (total genes encoded in nuclear, mitochondrial, and chloroplast genomes). DEGs with log2 FC (fold change) > 1 are shown as induced genes (magenta), and genes with log2 FC < −1 are shown as suppressed genes (green). B, C, Association between GO terms of Biological Process class (B) and Cellular Component class (C) for terms detected in all five mitochondrial dysfunction mutants.

As several mutants with various mitochondrial dysfunctions all exhibited an increase in the number of mitochondrial genomes (Figure 5), we conducted an RNA-seq analysis to investigate whether the expression of mitochondrial genes is also induced as observed in *slo3* and *nad7* KO. We calculated the proportion of genes that were differentially expressed between mutants and the wild-type in each genome. Substantially higher proportions of induced DEGs were observed for the mitochondrial genome in all 7 mutants (Figure 6A and Supplemental Figure 4). On the other hand, substantially lower proportions of repressed DEGs were observed for mitochondrial genome in 5 mutants except for *rpoTmp* (Figure 6A, Supplemental Table 5–12). This is reasonable because *RPOTmp* is an organelle RNA polymerase and the *rpoTmp* mutant is known to have lowered transcript abundance in multiple genes encoded by mitochondrial and chloroplast genome (Kühn et al., 2009). Taken together, these results suggest that the expression of mitochondrial genome is generally induced in the mutants with increased mitochondrial genome copy number.

In addition, gene ontology (GO) analyses of the DEGs whose expressions were induced in the five mitochondrial respiratory chain-defective mutants (*ndufv1*, *otp87*, *rpoTmp*, *rppr5*, and *wtf9*) revealed many GO terms shared by multiple mutants (Supplemental Table 13–18). Of 56 GO terms that were commonly enriched in all mutants, many were related to mitochondrial function and ribosome and protein translation (Figure 6 B, CTable 1). These GO terms included organellar large ribosomal subunit (GO:0000315) and mitochondrial ribosome (GO:0005761), which is involved in mitochondrial translation, and protein localization to mitochondrion (GO:0070585), which is involved in protein transport from the cytoplasm to the mitochondria. Also, mitochondrion organization (GO:0007005), involved in mitochondrial biogenesis and maintenance, was also included. A pathway analysis using Kyoto Encyclopedia of Genes and Genomes (KEGG) database showed that many genes involved in glycolysis (path: ath00010) and TCA cycle (path: ath00020) were commonly induced in these mutants (Supplemental Figure 5). These results suggest that not only mitochondrial genes but also nuclear genes functioning in mitochondria are globally induced in the mutants with increased mitochondrial genome copy number.

**Table 1:** List of common enriched GO terms detected in all five mutants

ID	Description	Detected^a^	Class^b^	Function^c^
GO:0000028	ribosomal small subunit assembly	5/5	BP	ribo
GO:0000470	maturation of LSU-rRNA	5/5	BP	ribo
GO:0002181	cytoplasmic translation	5/5	BP	ribo
GO:0006364	rRNA processing	5/5	BP	ribo
GO:0006414	translational elongation	5/5	BP	ribo
GO:0009098	leucine biosynthetic process	5/5	BP	
GO:0016072	rRNA metabolic process	5/5	BP	ribo
GO:0022613	ribonucleoprotein complex biogenesis	5/5	BP	ribo
GO:0022618	ribonucleoprotein complex assembly	5/5	BP	ribo
GO:0032787	monocarboxylic acid metabolic process	5/5	BP	
GO:0042254	ribosome biogenesis	5/5	BP	ribo
GO:0042255	ribosome assembly	5/5	BP	ribo
GO:0042273	ribosomal large subunit biogenesis	5/5	BP	ribo
GO:0042274	ribosomal small subunit biogenesis	5/5	BP	ribo
GO:0070585	protein localization to mitochondrion	5/5	BP	mito
GO:0070925	organelle assembly	5/5	BP	mito
GO:0071826	ribonucleoprotein complex subunit organization	5/5	BP	ribo
GO:0072655	establishment of protein localization to mitochondrion	5/5	BP	mito
GO:0000313	organellar ribosome	5/5	CC	ribo/mito
GO:0000315	organellar large ribosomal subunit	5/5	CC	ribo/mito
GO:0005730	nucleolus	5/5	CC	
GO:0005732	sno(s)RNA-containing ribonucleoprotein complex	5/5	CC	ribo
GO:0005740	mitochondrial envelope	5/5	CC	mito
GO:0005742	mitochondrial outer membrane translocase complex	5/5	CC	mito
GO:0005743	mitochondrial inner membrane	5/5	CC	mito
GO:0005746	mitochondrial respirasome	5/5	CC	mito
GO:0005747	mitochondrial respiratory chain complex I	5/5	CC	mito
GO:0005750	mitochondrial respiratory chain complex III	5/5	CC	mito
GO:0005758	mitochondrial intermembrane space	5/5	CC	mito
GO:0005759	mitochondrial matrix	5/5	CC	mito
GO:0005761	mitochondrial ribosome	5/5	CC	ribo/mito
GO:0005840	ribosome	5/5	CC	ribo
GO:0006626	protein targeting to mitochondrion	5/5	CC	mito
GO:0007005	mitochondrion organization	5/5	CC	mito
GO:0009505	plant-type cell wall	5/5	CC	
GO:0015934	large ribosomal subunit	5/5	CC	ribo
GO:0015935	small ribosomal subunit	5/5	CC	ribo
GO:0019866	organelle inner membrane	5/5	CC	mito
GO:0022625	cytosolic large ribosomal subunit	5/5	CC	ribo
GO:0022626	cytosolic ribosome	5/5	CC	ribo
GO:0022627	cytosolic small ribosomal subunit	5/5	CC	ribo
GO:0030684	preribosome	5/5	CC	ribo
GO:0030964	NADH dehydrogenase complex	5/5	CC	mito
GO:0031225	anchored component of membrane	5/5	CC	
GO:0031428	box C/D RNP complex	5/5	CC	
GO:0031966	mitochondrial membrane	5/5	CC	mito
GO:0031970	organelle envelope lumen	5/5	CC	mito
GO:0032040	small-subunit processome	5/5	CC	
GO:0044391	ribosomal subunit	5/5	CC	ribo
GO:0045271	respiratory chain complex I	5/5	CC	mito
GO:0045275	respiratory chain complex III	5/5	CC	mito
GO:0070469	respirasome	5/5	CC	ribo
GO:0098798	mitochondrial protein-containing complex	5/5	CC	mito
GO:0098800	inner mitochondrial membrane protein complex	5/5	CC	mito
GO:0098803	respiratory chain complex	5/5	CC	mito
GO:1990204	oxidoreductase complex	5/5	CC	mito

Number of mutants for each GO term detected in enrichment analysis.

GO term class; BP: Biological Process, CC: Cellular Component.

The general function of each GO term; mito: mitochondrial, ribo: ribosomal.

### Mutants with increased genome copy number also show increased mitochondrial particle number

**Figure 7:**
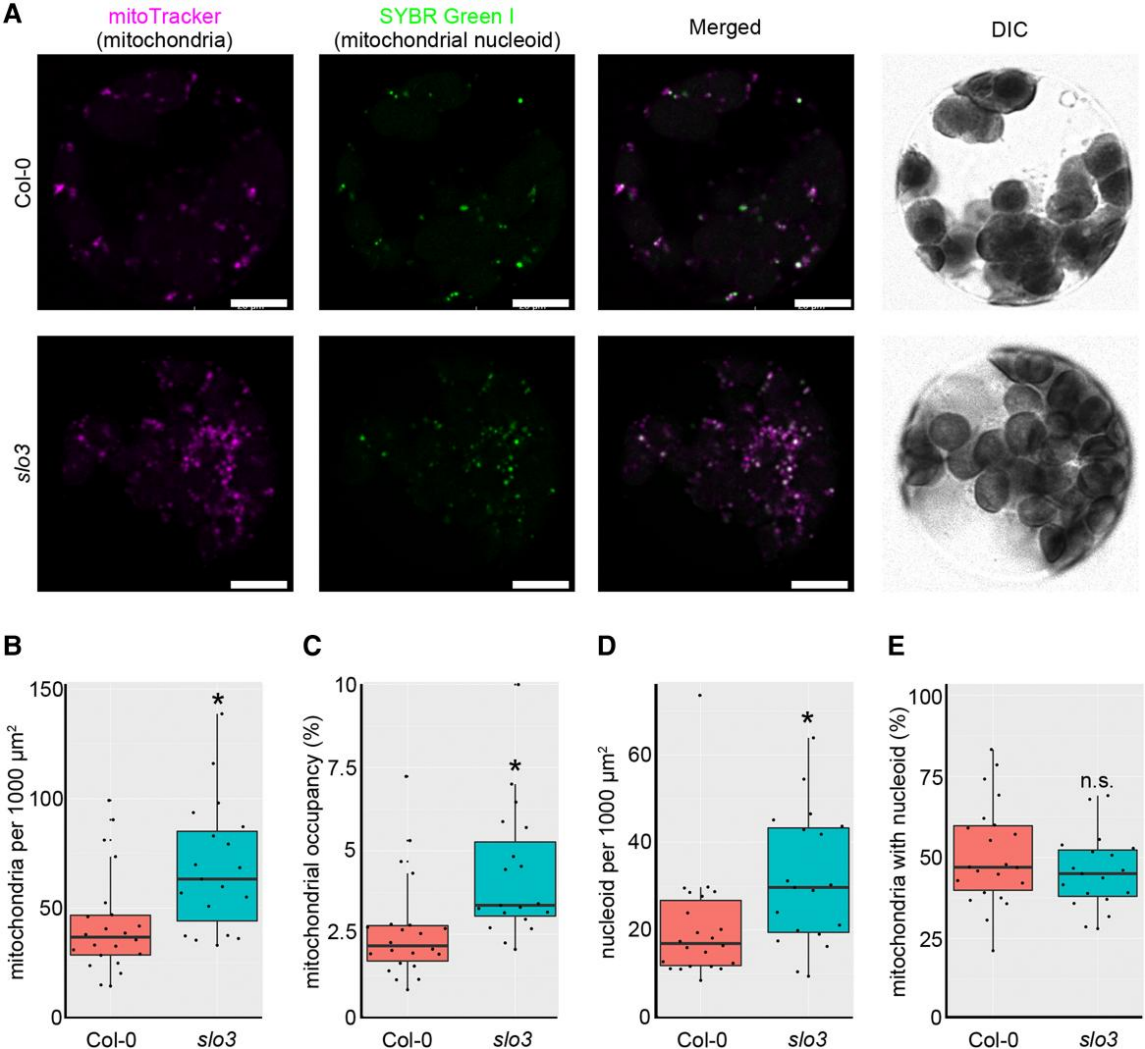
Mitochondrial morphology in *slo3*. A: Representative images of the protoplasts. From left to right: mitoTracker stain for mitochondria, SYBR Green I stain for nucleic acids, especially for mitochondrial nucleoids, merged image of two fluorescent images, and DIC (differential interference contrast). Scale bars = 10 µm. B–E, Protoplast analysis; number of mitochondrial particles per cell (B), mitochondrial area per cell (C), number of mitochondrial nucleoid bodies per cell (D), and percentage of mitochondrial particles with nucleoid (E). A total of 829 mitochondrial particles from 22 cells in the wild type and 1,601 mitochondrial particles from 19 cells in *slo3* were analyzed. Characteristics of the boxplots are similar to Figure 1G. * indicates statistical significance by Student's *t* test (*P* < 0.05).

The GO analysis suggested that the expression of genes involved in mitochondrial translation and biosynthesis was induced in mutants with increased mitochondrial genome copy number. To examine the numbers of mitochondrial particles and nucleoids, we prepared protoplasts of *slo3* and wild-type plants and then analyzed the structures of mitochondria with confocal laser scanning microscope (Figure 7A). The number of mitochondrial particles per 1,000 µm^2^ (Figure 7B), the percentage of the area occupied by mitochondria (Figure 7C), and the number of mitochondrial nucleoids per 1,000 µm^2^ (Figure 7D) were significantly higher in *slo3* than in the wild-type. On the other hand, there was no significant difference in the proportion of mitochondria with a nucleoid (Figure 7E). These results suggest that *slo3* mutants with increased mitochondrial genome copy number also have increased mitochondrial particle numbers.

### Association between mitochondrial genome copy number and gene expression

**Figure 8:**
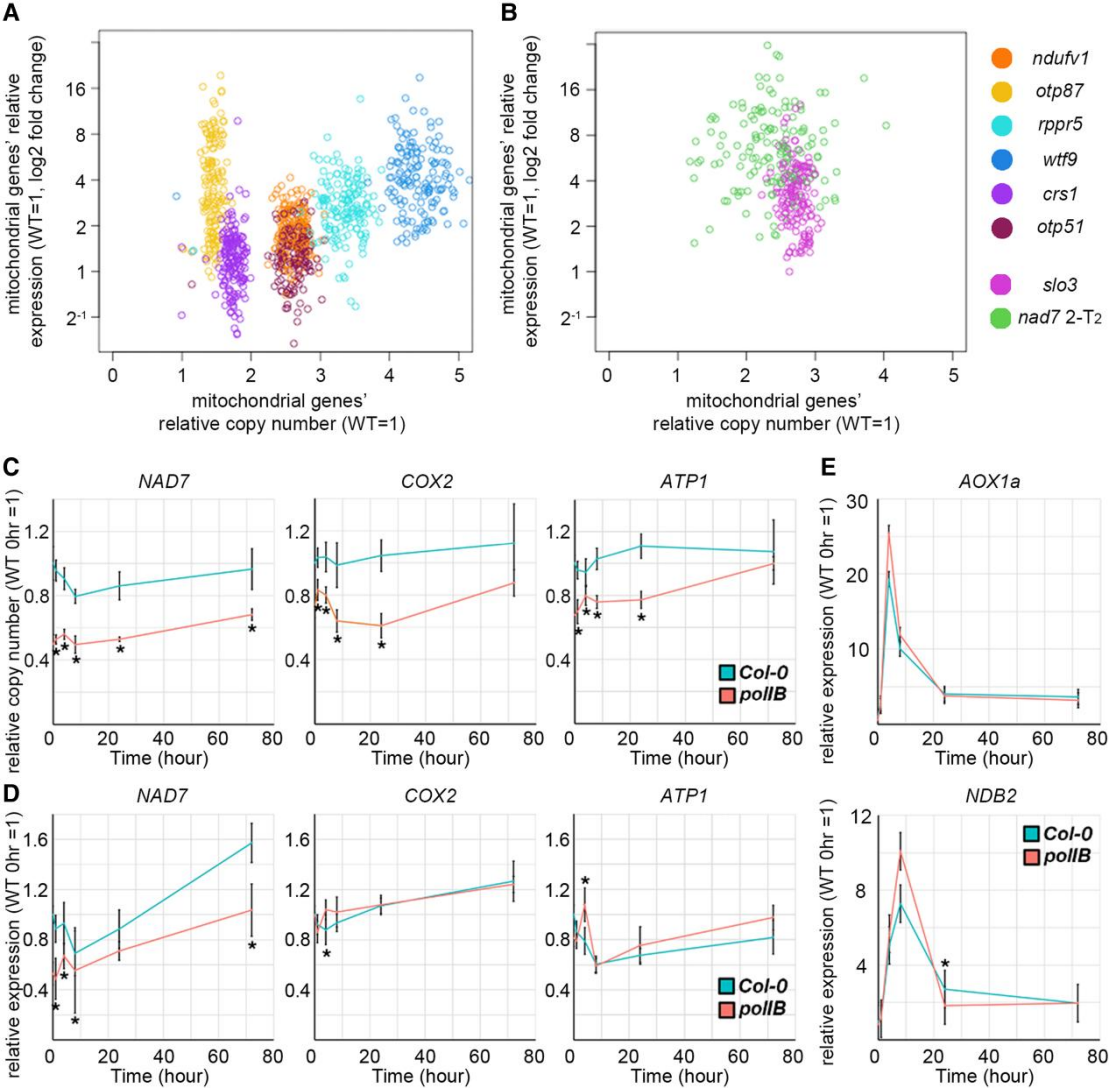
The relationship between mitochondrial genome copy number and expression under increased or decreased copy number. A, B, Relationship between mitochondrial genome copy number and transcript abundance. The horizontal and vertical axes are the relative copy number and transcript abundance of mitochondrial genes, respectively. Each point on the scatter plot corresponds to a gene in the organelle genome. C–E, The copy number (C) and expression (D, E) of mitochondrial genes and mitochondrial dysfunction responsive genes in *polIB* with transient AA treatment. Each gene was standardized by *TUA6,* and relative values (wild-type 0 hour as 1) are shown. Values are means ± SD of *n* = 5 for each time point. * indicates statistical significance by Student's *t* test (*P* < 0.05) between *polIB* and Col-0 at the same time point.

As stated above, we observed an increase in the copy number of the mitochondrial genome in several mutants and observed an increase in the gene expression of mitochondrial genes. In order to examine the relationship between copy number and expression level, we calculated the copy number and expression level of each gene in the organellar genome of the mutants from the NGS results and examined the association (Figure 8, A and B and Supplemental Figure 6; *rpoTmp* was omitted due to its transcriptional dysfunction). In seven of the eight mutants, a positive association was observed between copy number and expression level; as the mitochondrial genome copy number increase became more pronounced, an increase in gene expression was also observed (Figure 8, A and B). *otp87*, the mutant with the mildest growth retardation, did not have significantly more mitochondrial genomes than the wild-type (shown in Figure 6B). However, it contained more genes with higher transcript abundance than the other mutants. For genes in the chloroplast genome, no clear trend was observed in any of the mutants (Supplemental Figure 6).

Next, we examined the effect of a reduced mitochondrial genome copy number on gene expression. We used *poIIB*, a deletion mutant of a replication enzyme of the organelle genome, *Polymerase gamma 1*, which shows reduced mitochondrial and chloroplast genomes copy number (Parent et al., 2011). *polIB* and wild-type plants were sprayed with AA solution, and the copy number and expression levels of mitochondrial genes and nuclear-encoded mitochondrial stress responsive genes were analyzed over the following 72 hours. A significant decrease in copy number of *NAD7, Cytochrome oxidase 2*; *COX2* and *ATP synthase subunit 1*; ATP1 was observed compared with the wild-type (Figure 8C), as was shown in previous studies. For gene expression, no clear trend was observed for mitochondrial stress responsive genes; *AOX1a* and *NDB2* (Figure 8E), which suggests that nuclear gene expression in *polIB* can respond similarly to the wild-type. Mitochondrial *NAD7* showed a significant decrease in gene expression compared with the wild-type at all time points, a trend positively correlated with copy number. On the other hand, *COX2* and *ATP1* did not show such correlation in this condition (Figure 8, C and D). Taken together, these results suggest that the abundance of mRNA of some mitochondrial genes, at least *NAD7,* is positively correlated to the mitochondrial genome copy number, and other controls also exist.

### Mitochondrial genome copy number response to inhibitor treatment in other plant species

**Figure 9:**
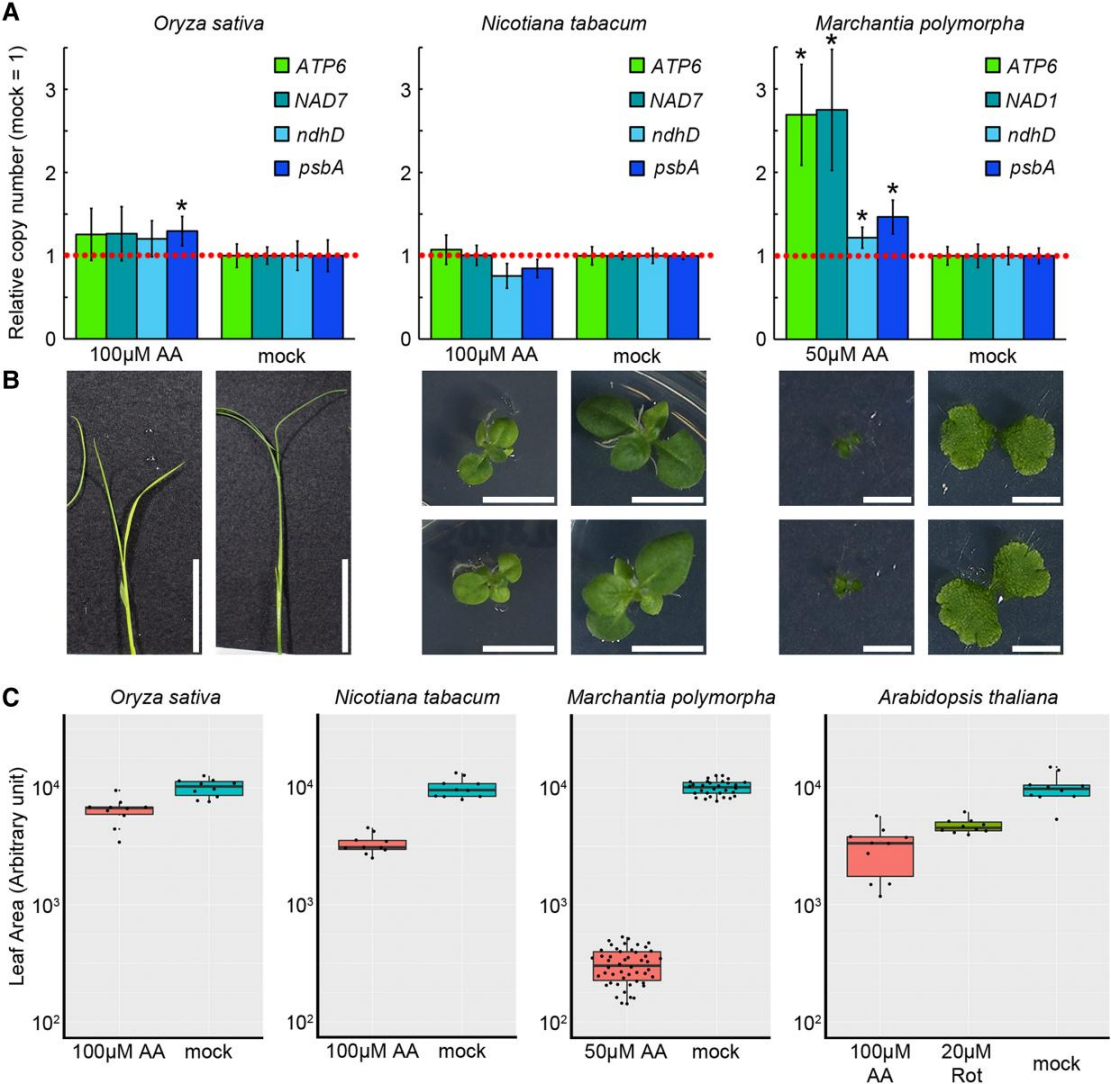
Copy number responses in other plant species. A–C, Quantification of organelle genome copy number (A), their phenotypes (B), and leaf areas (C) of inhibitor treated *Oryza sativa* (Nipponbare), *Nicotiana tabacum* (SR-1), and *Marchantia polymorpha* (Tak-1). Plants were grown for 7 days on AA (100 or 50 µM) containing, or inhibitor-free (mock) solid medium. Each gene was standardized by *TUA6,* and relative values (wild-type as 1) are shown. Characteristics of the boxplots are similar to Figure 1, G and 8B-8E. Values are means ± SD of *n* = 10 except for liverwort in C whose values are means ± SD of *n* = 60. * indicates statistical significance by Student's *t* test (*P* < 0.05) in (A). Scale bar = 4 cm in *Oryza sativa*, 1 cm in *Nicotiana tabacum*, and 5 mm in *Marchantia polymorpha*.

In Arabidopsis, mitochondrial genome copy number is increased under broad mitochondrial dysfunction and this increase appears to boost the abundance of transcripts of some mitochondrial genes. We were interested in whether this response is conserved in other plant species. To this end, we treated rice (*Oryza sativa* cultivar Nipponbare), tobacco (*Nicotiana tabacum* SR-1), and liverwort (*Marchantia polymorpha* Tak-1) with AA and quantified their organelle genome copy number. The phenotypes of treated or untreated plants at sampling are shown in Figure 9A. In rice and tobacco, no significant increase was observed. In contrast, a significant increase was observed in liverwort, as it was in Arabidopsis (Figure 9B). As the copy number increase was positively correlated with leaf area in Arabidopsis (Figure 5H), we calculated the relative leaf areas of each species (Figure 9C). Compared with Arabidopsis, rice showed a milder decrease in leaf area due to inhibitor treatment, tobacco showed no significant difference, and liverwort showed a more decrease. In other words, the similar degree of dysfunction causing a copy number increase in Arabidopsis could not induce the increase in tobacco in this condition. Taken together, these results suggest that liverwort, like Arabidopsis, shows an increase in mitochondrial genome copy number in response to mitochondrial dysfunction. While in rice and tobacco, such responses may be even absent or at least have different threshold for dysfunction inducing the copy number increase.

### Mitochondrial genome responses are partially regulated by nuclear stress-responsive factors, *ANAC017* and *RCD1*

**Figure 10:**
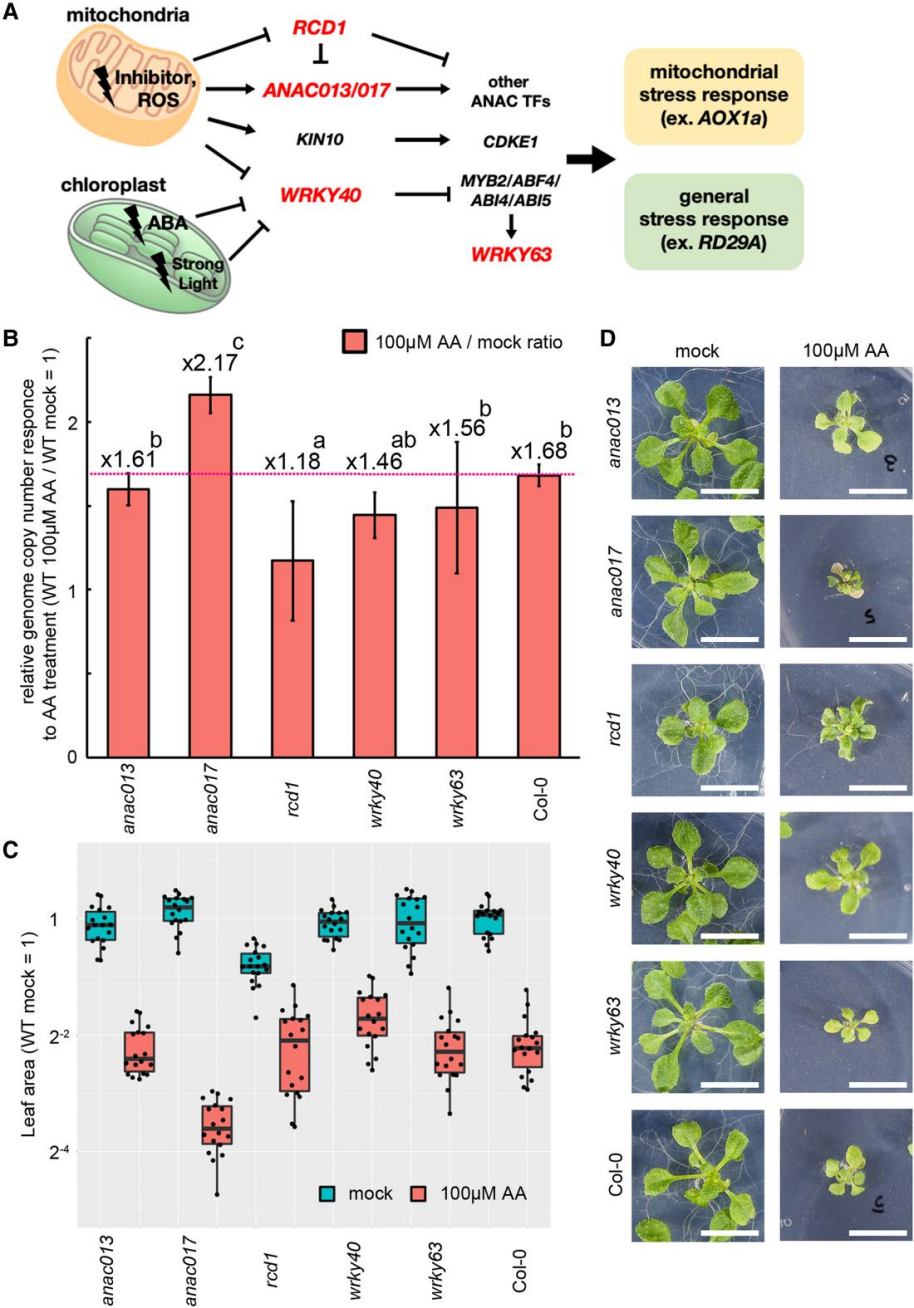
Screening of genes controlling mitochondrial genome responses. A, Schematic models of transcription factors involved in response to mitochondrial dysfunction. Loss-of-function mutants of the genes shown in red were used in the analysis. ROS, reactive oxygen species; ABA, abscisic acid; TF, transcription factors; KIN10, SNF1 kinase homolog 10; CDKE1, cyclin-dependent kinase E1; MYB2, MYB domain protein 2; ABF4, ABRE binding factor 4; ABI4/5, ABA insensitive 4/5. B–D, Mitochondrial genome copy number (B), leaf area (C) and their representative phenotypes (D) of the mutants. Plants grown for 14 days in solid medium containing Antimycin A (100 µM) and no inhibitors (mock) were used. B, Each gene was standardized by *TUA6,* and relative values (wild-type as 1) are shown. Values are means ± SD of *n* = 18 from independent experiments. Characteristics of the boxplots are similar to Figure 1, G, 8, B–E, and 9C. The different letters indicate significant differences between groups by a one-way ANOVA with post hoc Tukey's test (*P* < 0.05). D, All scale bars are 1 cm.

To further gain insight about factors triggering or controlling mitochondrial genome copy number response, we treated mutants of mitochondrial stress responsive genes (*NAC domain containing protein 13*; *anac013*, *NAC domain containing protein 17*; *anac017*, *Radical cell death 1*; *rcd1*, *WRKY DNA binding protein 40*; *wrky40*, and *WRKY DNA binding protein 63*; *wrky63*) with AA (Figure 10A). In these 5 mutants, *rcd1* and *anac017* showed mild but significant hypo- and hyper-sensitivity to AA treatment, respectively (Figure 10, B–D). These genes were reported as repressor and activator of mitochondrial stress response, respectively (Ng et al., 2013; Shapiguzov et al., 2019). Therefore, this result suggests that the increase in mitochondrial genome copy number under mitochondrial dysfunction is at least partially under the control of *RCD1* and *ANAC017*.

## Discussion

### Disruption of Arabidopsis *NAD7* gene results in severe growth delay different from *Nicotiana sylvestris* CMSII

We attempted to disrupt the *NAD7* gene using the mitoTALEN method. Among the 30 T_1_ transformants produced, one strain (2-T_2_) was expected to be a *nad7* KO strain. This line showed marked growth retardation as well as a mitochondrial stress response that eventually resulted in the lethality of all plants (Figure 1). In the 5.5 kb region deleted from the mitochondrial genome of 2-T_2_, there were no genes other than *NAD7* except for two overlapped ORFs (ORF141 and ORF107C) with unknown functions. BLAST searches of these ORFs’ sequences detected homology at the amino acid level with the RNA-dependent RNA polymerase of RNA viruses, suggesting that it is unlikely to be involved in the ordinary mitochondrial function. Therefore, the phenotypes observed in 2-T_2_ probably originated from the loss of *NAD7*, and its complete deletion could be lethal in Arabidopsis. This is contrasting to *N. sylvestris nad7* KO mutant *CMSI* and *II*, with viable and CMS phenotypes. This difference could be due to incomplete deletion of *NAD7* in *CMSI* and *II* (Lelandais et al., 1998).

Various mitochondrial dysfunction induces mitochondrial genome copy number increase and general increased accumulation of mRNAs in *A. thaliana*.

Some mutants with mitochondrial dysfunctions were reported to have increased mitochondrial genome copy number (*rps10*Kuwasniak et al., 2013; *rpoTmp*Kühn et al., 2009; *shot1*Kim et al., 2012), but these reports are sporadic and the trigger, the universality, or the impact of this phenomenon were not deeply analyzed. Here, we observed mitochondrial genome copy number increase per cell and increased accumulation of mRNA in seven mutants, two respiratory inhibitors, and high light conditions. These included complex I dysfunction (*nad7* KO line, *slo3*, *ndufv1*, and Rotenone treatment), complex III dysfunction (AA treatment), and complex IV dysfunction (*wtf9*), a wide range of effects on mitochondrial gene transcription (*rpoTmp*) and translation (*rppr5*), photosystem II dysfunction (*otp51*), and high light conditions that require mitochondrial functions such as photorespiration and relaxation of hyper-reduction (Noguchi and Yoshida, 2008). Moreover, the copy number increase was positively correlated with the degree of growth retardation and the expression level of *AOX1a*, a marker gene for stress response against mitochondrial dysfunction (Figure 5). Therefore, it is unlikely that the increase is only triggered by a lack of transcripts, proteins, or complexes derived from specific genes, and it is more likely that the increase is induced by universal factors that increase in abundance under mitochondrial dysfunction. In the nuclear gene expression response to mitochondrial dysfunction, reactive oxygen species (ROS) and various molecules such as Ca^2+^ and intermediate metabolites have been reported to mediate retrograde signaling (de Souza et al., 2017; Crawford et al., 2018). Based on this, it is expected that similar molecules will act as messengers to induce mitochondrial genome copy number increase and increased accumulation of mRNA described here. In line with this hypothesis, the mitochondrial response was partially regulated by transcription factors controlling nuclear stress response (Figure 10). This scenario is consistent with the proposed model for coordinated expression of two genomes, nuclear and mitochondrial genome (Woodson and Chory, 2008).

### Mitochondrial genome copy number increase may be one of the factors that positively regulate the expression level of the mitochondrial genes

In this study, we observed a positive correlation between mitochondrial genome copy number and the overall expression level of the mitochondrial genes among Arabidopsis mutants (Figure 8A), while the mildest mutant *otp87* did not follow this relationship. For the regulation of mitochondrial gene expression, it has been considered to be mainly at the post-transcriptional stage and not strictly at the transcriptional stage (Kuwasniak et al., 2013). Moreover, there have been several contradictory reports on the relationship between copy number and expression level with mitochondrial dysfunction (Kühn et al., 2009; Preuten et al., 2010; Kuwasniak et al., 2013) and with rearranged mitochondrial genomes (Woloszynska et al., 2006; Shedge et al., 2010). The present results suggest that, in the presence of mitochondrial dysfunction in Arabidopsis, mitochondrial genome copy number is at least partially associated with the total gene expression of the mitochondria. However, it is unclear whether this association includes mutants with rearranged mitochondrial genomes, as the effects of the rearrangements on gene expression are difficult to evaluate. Interestingly, our data showed no substantial induction of genes involved in mitochondrial genome replication and transcription in the mutants with increased genome copy number and accumulation of transcripts (Supplemental Figure 7). One possible explanation for this is that the increase of these transcripts wasn’t needed to account for the increase in copy number or transcripts at the sampling stages. Other possibilities are that some post-transcriptional regulation might have occurred, or the response to the dysfunctions might have completed so that differential expression would not have been detected.

On the other hand, our analysis of mutants with reduced copy numbers showed a different relationship among the genes, and the copy number-expression association was confirmed for *NAD7,* but not for the copy numbers in COX2 or ATP1 or their gene expressions (Figure 8, C and D). This implies the existence of gene-specific regulation of expression, even though mitochondrial expressional regulation is not as strict as it is in the nuclear genome (Binder et al., 2003; Kühn et al., 2005). Taken together, these results suggest that broad mitochondrial dysfunction may have an overall effect on mitochondrial transcriptional profiles through copy number, and that a similar phenomenon exists at least in the liverwort (Figure 9). As mitochondrial transformation of angiosperms is expected to become more and more common, researchers should be aware of the possibility that a single mutation may affect the expression of the entire mitochondrial gene.

## Materials and methods

### Plant materials and growth conditions

Seeds of Arabidopsis (*Arabidopsis thaliana* ecotype: Col-0) were put on Jiffy-7 pots (Jiffy, http://www.jiffypot.com) or × 1/2 Murashige and Skoog (MS) agar plates and stratified at 4°C in darkness for 4 days before they moved to growth rooms. Arabidopsis plants were grown on soil or MS medium under long days (16-h light/8-h dark), illuminated by white fluorescent light or white light-emitting diodes (50–100 µmol/m^2^ sec, at 22°C). The mutants of *Slow growth 3; slo3* (SAIL_1242_B03), 5*1 kDa subunit of complex I*; *ndufv1* (SAIL_319_D07), *What this factor 9*; *wtf9* (SALK_022250), O*rganellar transcript processing 87*; *otp87* (GABI_073C06), P*hage-type RNA polymerase*; *rpoTmp* (SALK_132842), *Ribosomal pentatrico peptide repeat protein 5*; *rppr5* (SAIL_1146_C06), *Chloroplast splicing factor 1*; *crs1* (SALK_026861), *Organellar transcript processing 51*; *otp51* (SALK_112013), *Polymerase gamma 1*; *polIB* (SALK_134274C), NAC domain containing protein 13*; anac013* (SALK_09615°C), and *Radical cell death 1*; *rcd1* (CS9354) were obtained from the Arabidopsis Biological Resource Center (https://abrc.osu.edu/). *NAC domain containing protein 17; anac017* (SALK_022174), *WRKY DNA binding protein 40*; *wrky40* (CSHL_ET5883), *WRKY DNA binding protein 63*; *wrky63* (SALK_007496) were kindly provided by Dr. Olivier Van Aken (Lund University, Sweden). All the mutants analyzed in this paper were in the Col-0 background. And Arabidopsis was transformed with the mitoTALEN expression vectors by floral dipping (Clough and Bent, 1998). Rice (*Oryza sativa* Nipponbare) were put on x 1/2 MS agar plates and stratified at 4°C in darkness for 4 days before they moved to growth rooms. *Oryza sativa* were grown on soil or MS medium under long days (16-h light/8-h dark), illuminated by white fluorescent light (700 µmol/m^2^sec, at 26°C). Tobacco (*Nicotiana tabacum* SR-1) were put on × 1/2 MS agar plates and were grown under long days (16-h light/8-h dark), illuminated by white fluorescent light (50 µmol/m^2^sec, at 26°C). *Marchantia polymorpha* (Tak-1) were planted on × 1/2 Gamborg-B5 plates and were grown under long days (16-h light/8-h dark), illuminated by white fluorescent light (50 µmol/m^2^ sec, at 22°C). Tak-1 were kindly provided by Dr. Kiminori Toyooka (Kyoto University).

### Accession numbers

Sequence data from this article can be found under following accession numbers in the TAIR library for Arabidopsis genes and Uniprot database for other plants: AtNDUFV1 (AT5G08530), AtWTF9 (AT2G39120), AtOTP87 (AT1G74600), AtPolIA (AT1G50840), AtPolIB (AT3G20540), AtRpoTmp (AT5G15700), AtrPPR5 (AT2G37230), AtCRS1 (AT5G16180), AtOTP51 (AT2G15820), AtSLO3 (AT3G61360), AtNDA2 (AT2G29990), AtNDB2 (AT4G05020), AtAOX1a (AT3G22370), AtNAD7 (ATMG00510), AtCOX2 (ATMG00160), AtATP6-1 (ATMG00410), AtATP6-2 (ATMG01170), AtpsbA (ATCG00020), AtndhD (ATCG01050), NtUBC2 (F1C959), NtEF1a (P43643), NtATP6 (P05499), NtNAD7 (Q36450), NtpsbA (P69556), NtndhD (Q33BX2), OsACT (Q10DV7), OsTUB (P45960), OsATP6 (Q8S1Q3), OsNAD7 (Q8HCQ3), OspsbA (P0C434), OsndhD (P0C325), MpACT1 (Q852Q7), MpTUB8 (A0A0A7H9F7), MpNAD1 (P26845), MpATP6 (P26853), MppsbA (P06402), and MpndhD (P06263).

### Selection of target sequence for mitoTALENs and mitoTALEN expression vector construction

Target sequences of mitoTALENs were selected as described previously (Kazama et al., 2019). The actual DNA binding domains of each mitoTALEN is shown in Supplemental Figure S8. The expression vectors of mitoTALENs were constructed as described previously (Kazama et al., 2019). In total, we made 4 mitoTALEN expression vectors with a combination of 2 TALEN-left and -right motifs with different recognition sites. DNA recognition motifs for the selected target sequences were assembled with a Platinum Gate TALEN kit (Sakuma et al., 2013) into the entry vectors with one of the pairs of TALEN ORF (referred as right or left). Then, 2 entry vectors with TALEN right and left ORF and an additional entry vector for expression of TALEN ORF (with the same terminator, promoter and mitochondrial presequence as the destination vector) were transferred into the Gateway destination vectors of Ti-plasmids containing the promoter (RPS5Apro) and the mitochondrial presequence by multi-LR reactions (ThermoFisher Scientific, https://www.thermofisher.com). The additional vector was cloned into the destination vector between the right and left regions of the TALEN ORFs. The basic backbones of destination vectors are from pK7WG2 (Karimi et al., 2002). The TALEN ORF, promoter, terminator, and mitochondrial presequence was described previously (Arimura et al., 2020).

### DNA and RNA extraction

DNA and RNA of T_1_ mitoTALEN transformants were extracted from leaves of plants grown for 3 weeks after vernalization to obtain their progeny. DNA and RNA from the other plants were extracted from whole above-ground plant tissue of plants grown for 3 or 4 weeks after vernalization. For extraction, the samples were frozen with liquid nitrogen and then crushed with metal beads using a multi-bead shocker (Yasui Instrument, http://www.yasuikikai.co.jp/) at 1800 rpm for 30 s. To examine the relationship between DNA and RNA results, the crushed powder was divided into two parts and used for DNA and RNA extraction. Total DNA was extracted with DNeasy Plant Mini Kit (Qiagen, https://www.qiagen.com) for whole-genome sequencing analysis, and with Maxwell RSC Plant DNA kit (Promega, https://www. promega.com) for multiplex and qPCR analysis. Total RNA was extracted using Maxwell RSC Plant RNA kit (Promega). All extractions were performed according to the manufactures’ instruction.

### PCR analyses

Multiplex PCRs were carried out with KOD One® PCR Master Mix (TOYOBO, https://www.toyobo-global.com/). The primers and their combinations shown in Supplemental Table S19. The multiplex PCRs included 28 cycles of 94°C for 10 sec, 60°C for 5 sec and 68°C for 10 sec, below saturated amplifications. Quantitative PCR was carried out with TB Green® Premix Ex Taq™ GC (Perfect Real Time) (TaKaRa, https://www.takarabio.com) for DNA, and One Step TB Green® PrimeScript™ RT-PCR Kit II (TaKaRa) for RNA, using Step One Plus Real Time PCR System (ThermoFisher Scientific). The organellar genome copy number and their expression analysis were calculated from the difference between the cycle threshold (Ct) of organelle genes and the Ct of nuclear gene (ΔCt = Ct_organelle_ − Ct_control_), and the relative value (normalized to wild-type or mock as 1) were shown in figures. The primers used for qPCR analysis is shown in Supplemental Table S19.

### WGS analysis

Total DNA was used to prepare 350-bp libraries for the analyses shown in Figures 1–3 and 5 and 7. *nad7*, KO, *slo3*, and wild-type (Figures 1–3) were sequenced by, MacroGen Japan Inc. (https://www.macrogen-japan.co.jp), and paired-end sequencing was performed using a next-generation sequencer Novaseq6000 (Illumina) reading 150 bp at both ends. The library was prepared using the Nextera XT DNA Library Kit (Illumina) for *nad7* KO individuals as their low DNA content and wild-type DNA for comparison, and TruSeq DNA PCR Free (350) (Illumina) for *slo3* and wild-type DNA for comparison. Analysis of the other mutants (Figure 5) was commissioned to Dr. Atsushi Toyoda (National Institute of Genetics) through the Advanced Genome Support Program (https://www.genome-sci.jp) funded by the Ministry of Education, Culture, Sports, Science and Technology (MEXT). Paired-end sequencing was performed using a Novaseq 6000 (Illumina) next-generation sequencer reading 150 bp. TruSeq DNA PCR Free (350) (Illumina) was used for library preparation.

Deleted regions in 2-T_2_ by mitoTALEN were examined using Geneious Prime (Biomatters, https://www.geneious.com). First, any reads mapped to the plastid genome sequence (AP000423) were removed from the total reads to avoid confusing analysis of the mitochondrial genome. Then the remaining reads were mapped to the mitochondrial genome sequence of *Arabidopsis thaliana* Col-0(BK 010421). The copy number of the organelle genome and the organelle-encoded genes was estimated by dividing the number of total reads mapped to region of interest by the total reads mapped to the entire nuclear genome. The relative values of the normalized values (wild-type as 1) are shown in Figures 2 and 6. For analysis of organelle genome coverage variation, the number of reads mapped within a slide window of 500 bp through the mitochondrial genome is counted by samtools package in R. The coverage is calculated using CPM (counts per million mapped reads) normalized counts, and relative values (wild-type as 1) for each slide window are shown in Figure 2 and 3s.

### RNA-seq analysis

Total RNA was used to prepare 350-bp libraries for the analyses shown in Figures 4 and 7 and 8. For the library preparation, TruSeq Stranded Total RNA with Ribo-Zero Plant (Illumina) was used to analyze the transcription of the organelle genome, which has been suggested to contain transcripts without poly-A modification. 2-T_2_, *slo3*, and wild-type (Figure 4) were sequenced by MacroGen Japan Inc. and paired-end sequencing was performed using a next-generation sequencer Novaseq 6000 (Illumina) reading 100 bp at both ends. Analysis of the other mutants (Figure 6) was outsourced to Advanced Genome Support, and paired-end sequencing was performed using the next-generation sequencer Novaseq 6000 (Illumina) reading 100 bp at both ends. The obtained raw data were trimmed using trim_galore, mapped to the Arabidopsis reference genome (TAIR10) using HISAT2, and calculated as gene-by-gene count data using stringtie. The count data were normalized using the DESeq2 package in R to detect DEGs. Genes with an absolute value of log2 fold change greater than 1 and false discovery rate less than 0.05 were detected as DEGs. The obtained DEGs were subjected to GO analysis using goProfiles package in R.

### Phenotypic analysis

Leaf area was calculated using ImageJ based on photographs taken from above with a camera (EM-5, Olympus), or with stereomicroscope (Leica M125, Leica) equipped with camera (Leica MC170 HD, Leica) for smaller individuals.

### Flow cytometry analysis

For flow cytometry, whole above-ground parts of plants 3 or 4 weeks after vernalization were chopped with a razor blade in Quantum Stain NA UV 2 A solution (Cytotechs Inc., http://www.cytotechs.com/). The nuclei were filtered through a FiltriX filter (20 µm) and stained Quantum Stain NA UV 2 B solution containing 4,6- diamidino-2- phenylindole. The nuclear DNA content was analyzed with Ploidy Analyzer PA (Sysmex-Partec, https://www.sysmex-partec.com/) according to Kikuchi and Iwamoto (2020).

### Preparation of protoplasts and microscopic observation

Protoplasts were prepared according to Yoo et al (2007). The youngest fully expanded leaves (3rd or 4th true leaf for Col-0 and 1st or 2nd true leaves for *slo3*) were used. Protoplasts were stained with 1/2,000 dilutions of SYBR Green I (TaKaRa, https://www.takarabio.com) and 1 µM mitoTracker Orange CMTMRos (ThermoFisher Scientific). Protoplasts were observed using a Leica STELLARIS5 confocal microscope (Leica) with a HC PL APO CS2 20× DRY objective lens (N.A. = 0.75) and HyD detectors. The Leica STELLARIS5 microscope was controlled by LAS × software (Leica), SYBR Green I and mitoTracker Orange CMTMRos were excited with 488 and 561 nm tuned white light laser, respectively. The fluorescence emissions were detected through a band-pass filter at 503–570 nm with 6.25% gain and a filter at 583–640 nm with 1.25% gain for SYBR Green I and mitoTracker Orange CMTMRos, respectively. The obtained images were analyzed by Image J for mitochondria and nucleoids.

### Statistical analysis

All statistical analyses were performed by R version 4.1.0. The significance analysis was determined by the one-way ANOVA followed by post hoc Tukey's test in “multcomp” package or Student's *t* test in “stats’ package. *P* < 0.05 was considered the significant difference.

## Supplemental data

The following materials are available in the online version of this article.

**Supplemental Figure S1.** Structure of repaired mitochondrial genome in *nad7* 2-T_2_.

**Supplemental Figure S2.** Relative coverage variation in mitochondrial genome of mutants analyzed in Figure 5.

**Supplemental Figure S3.** Ploidy measurements in mutants with increased mitochondrial genome copy number.

**Supplemental Figure S4.** Transcriptional profiles in mutants with chloroplast dysfunctions.

**Supplemental Figure S5.** Pathway analysis of glycolysis (path: ath00010) and TCA cycle (path: ath00020).

**Supplemental Figure S6.** Relationship between plastid genome copy number and expression in plastid genes.

**Supplemental Figure S7.** Heatmaps of mitochondrial DNA replication and RNA transcription genes.

**Supplemental Figure S8.** The target sequence of mitoTALENs in *NAD7* gene.

**Supplemental Table S1.** The actual number of DEGs detected for each genome in the *nad7* 2-T_2_ and *slo3*.

**Supplemental Table S2.** List of DEGs detected in *nad7* 2-T_2_ against wild-type.

**Supplemental Table S3.** List of DEGs detected in *slo3* against wild-type.

**Supplemental Table S4.** Details of mutants.

**Supplemental Table S5.** The actual number of DEGs for each genome detected in the mutants analyzed in Figure 5.

**Supplemental Table S6.** List of DEGs detected in *ndufv1* against wild type.

**Supplemental Table S7.** List of DEGs detected in *otp87* against wild type.

**Supplemental Table S8.** List of DEGs detected in *rpoTmp* against wild type.

**Supplemental Table S9.** List of DEGs detected in *rppr5* against wild type.

**Supplemental Table S10.** List of DEGs detected in *wtf9* against wild type.

**Supplemental Table S11.** List of DEGs detected in *cr1* against wild type.

**Supplemental Table S12.** List of DEGs detected in *otp51* against wild type.

**Supplemental Table S13.** List of GO terms detected in *ndufv1*.

**Supplemental Table S14.** List of GO terms detected in *otp87*.

**Supplemental Table S15.** List of GO terms detected in *rpoTmp*.

**Supplemental Table S16.** List of GO terms detected in *rppr5*.

**Supplemental Table S17.** List of GO terms detected in *wtf9*.

**Supplemental Table S18.** Table of differentially expressed genes (DEGs) in 56 common GO terms in Table 1.

**Supplemental Table S19.** Primer list.

## Supplementary Material

kiad024_Supplementary_Data
