# Supplementary material for: Mitochondrial gene defects in Arabidopsis can broadly affect mitochondrial gene expression through copy number
Source: Plant Physiol. 2023 Jan 27;191(4):2256–75. doi: 10.1093/plphys/kiad024 (PMC10069900; doi:10.1093/plphys/kiad024)
Supplement: kiad024_Supplementary_Data [file kiad024_supplementary_data.zip › 230104 Supplemental Figures HA.pdf]

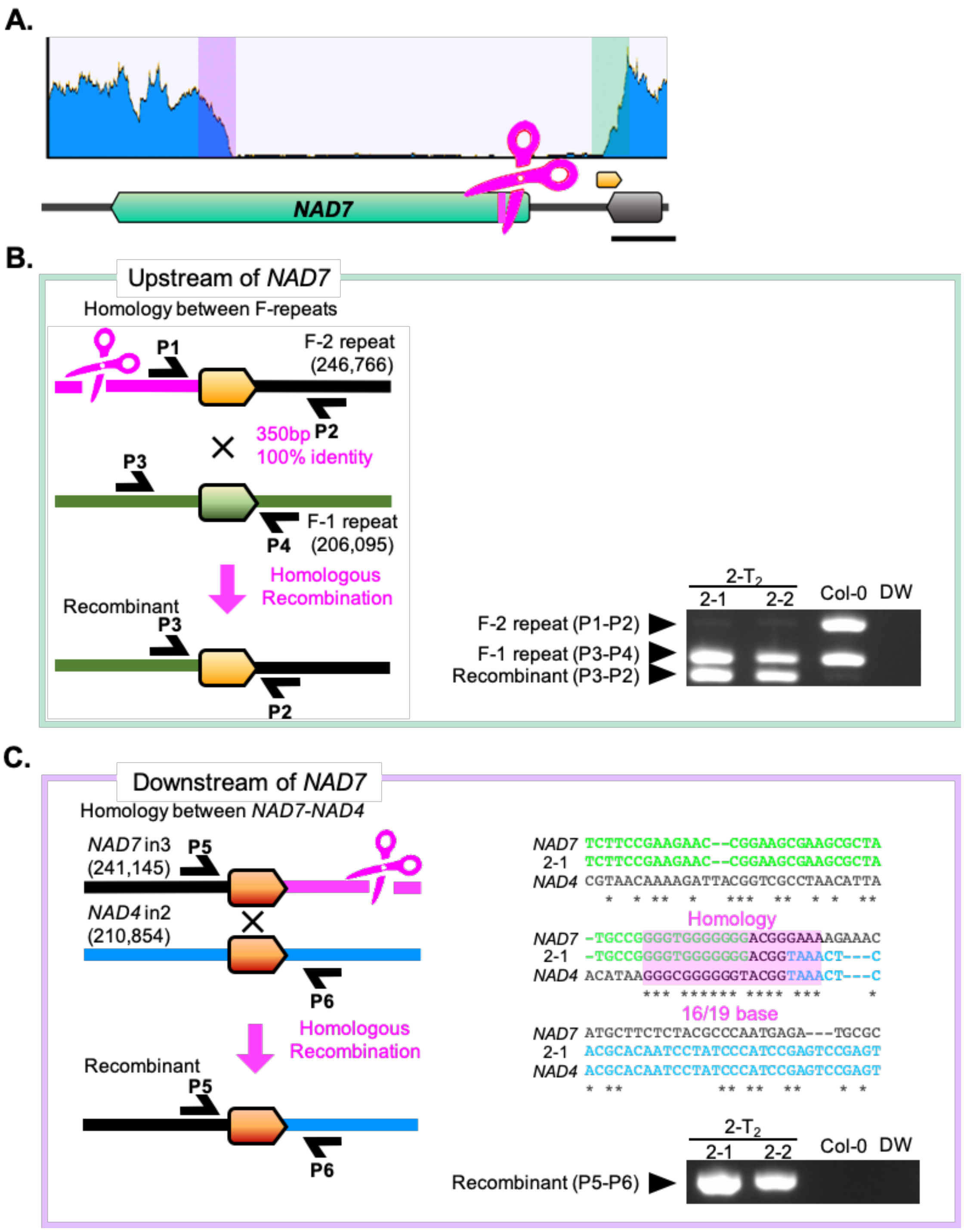

Supplemental Figure 1

Structure of repaired mitochondrial genome in *nad7* 2-T_2.

A: Schematic drawing of mitochondrial genome around the target. Green and purple boxes correspond to up- and downstream region shown in B and C.

B, C: Repaired structure in *nad7* 2-T_2. Homology used for recombination repair

and results of recombinant detecting PCR were shown. Arrows indicate the primer positions used for PCR. These primers were simultaneously reacted, and Recombinant sequence could be detected. Sequence in C shows recombinant sequence in *nad7* 2-1. Each lane corresponds to *NAD7*, *nad7* 2-1 recombinant, *NAD4* from top to bottom. Green or blue letters show identity between recombinant and *NAD7* or *NAD4*, respectively. Red box indicates estimated homology used for homologous recombination in *nad7* 2-1.

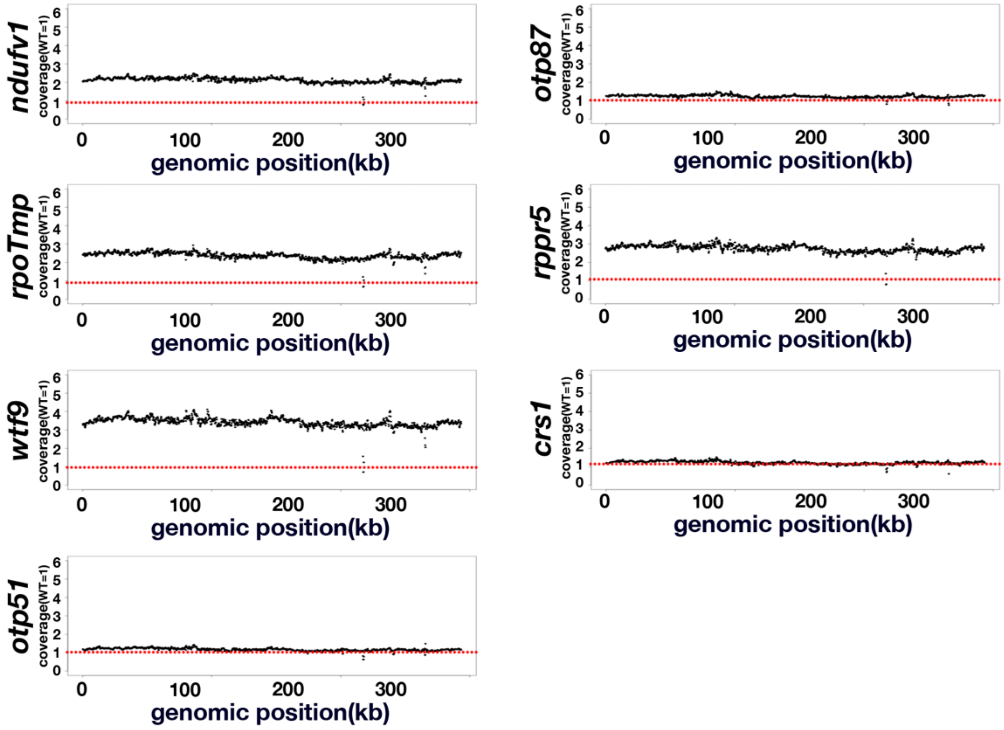

Supplemental Figure 2

Relative coverage variation in mitochondrial genome of mutants analyzed in Figure 5. Relative values (wild type as 1) are shown. Horizontal red dotted line indicate the average wild type's copy number for each genomic region.

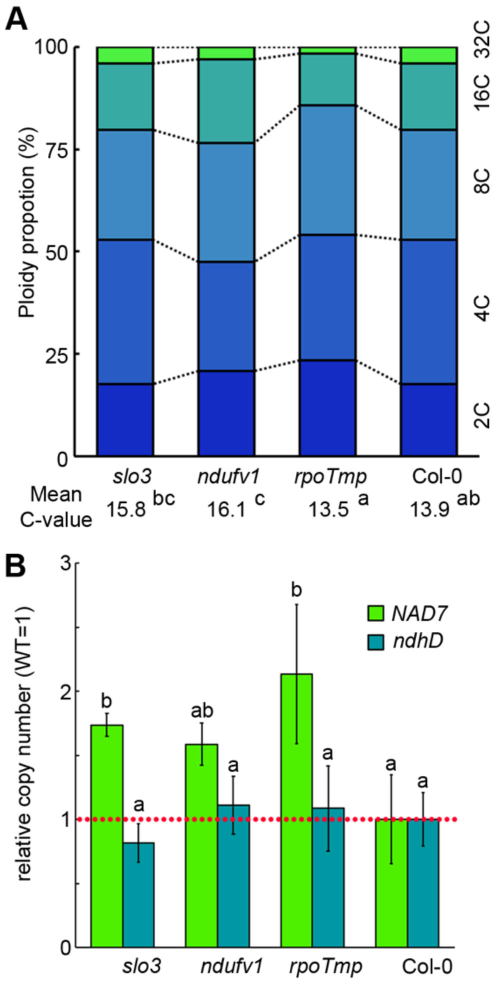

Supplemental Figure 3

A: Nuclear ploidy measured by flow cytometer. Measurements were performed on more than 1000 nuclei from each sample of plants 3 weeks after vernalization. Values are means of n=4.

B: Organelle genome copy number of the plants whose ploidy were measured in (A). Each gene was standardized by *TUA6*, and the estimated values were corrected by C-value. Relative values (wild type as 1) are shown. Values are means ± SD of n = 4. The different letters indicate significant differences between groups by a one-way ANOVA with post hoc Tukey’s test ($P < 0.05$).

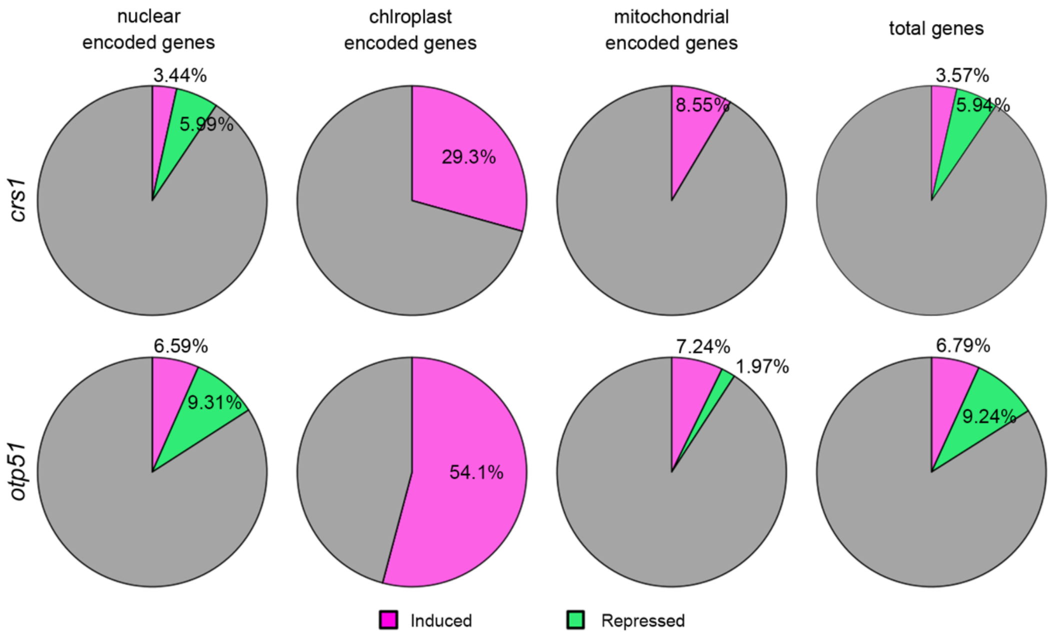

Supplemental Figure 4.

Transcriptional profiles in mutants with chloroplast dysfunctions

The proportion of DEGs against total genes encoded in each genome. From left to right, DEGs proportion among nuclear-encoded genes, mitochondrial-encoded genes, plastid-encoded genes, and the total genes (total genes encoded in nuclear, mitochondrial, and chloroplast genomes). DEGs with log2 FC (fold change) > 1 are shown as induced genes (magenta), and genes with log2 FC < -1 are shown as suppressed genes (green).

A ***ndufv1***

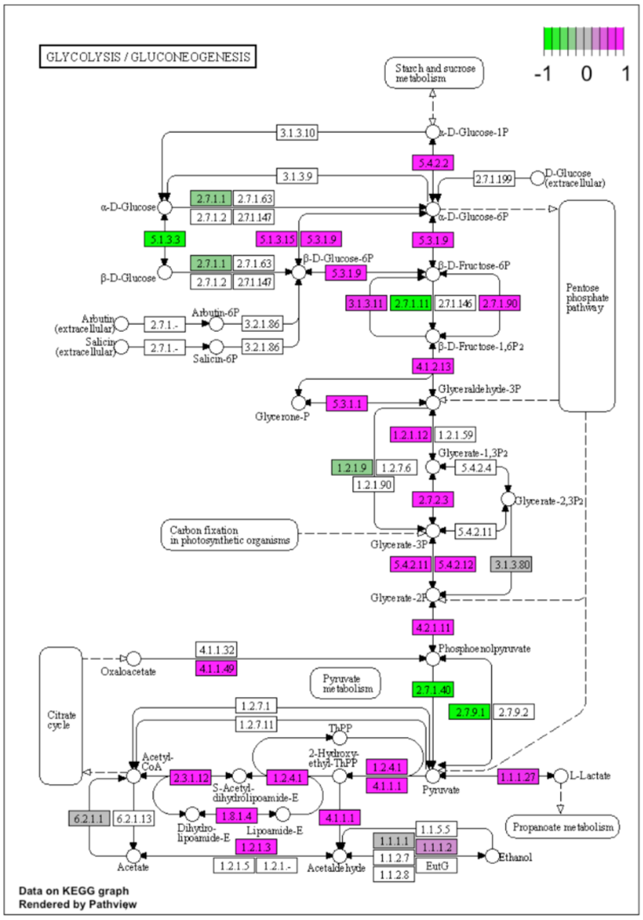

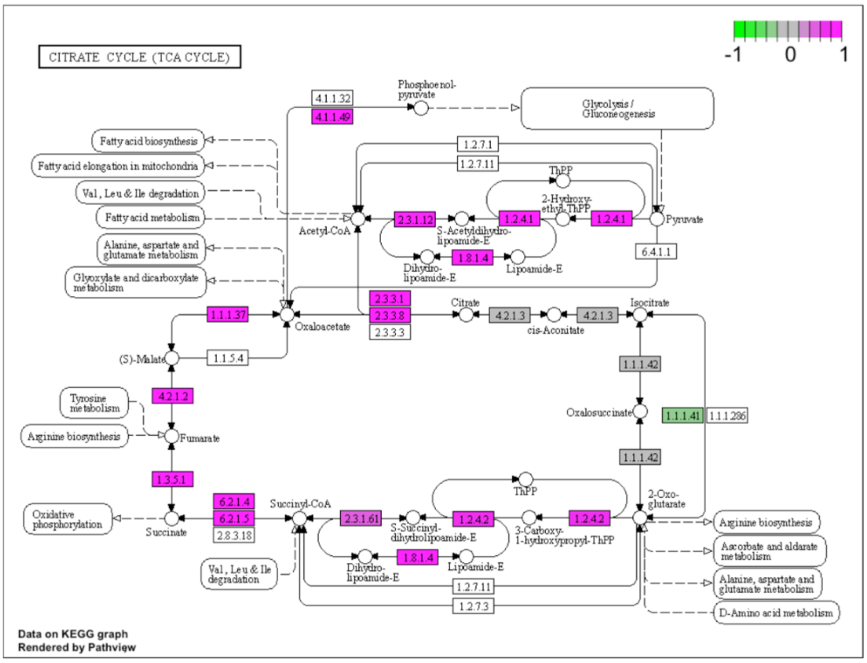

Supplemental Figure 5 (continued)

B ***otp87***

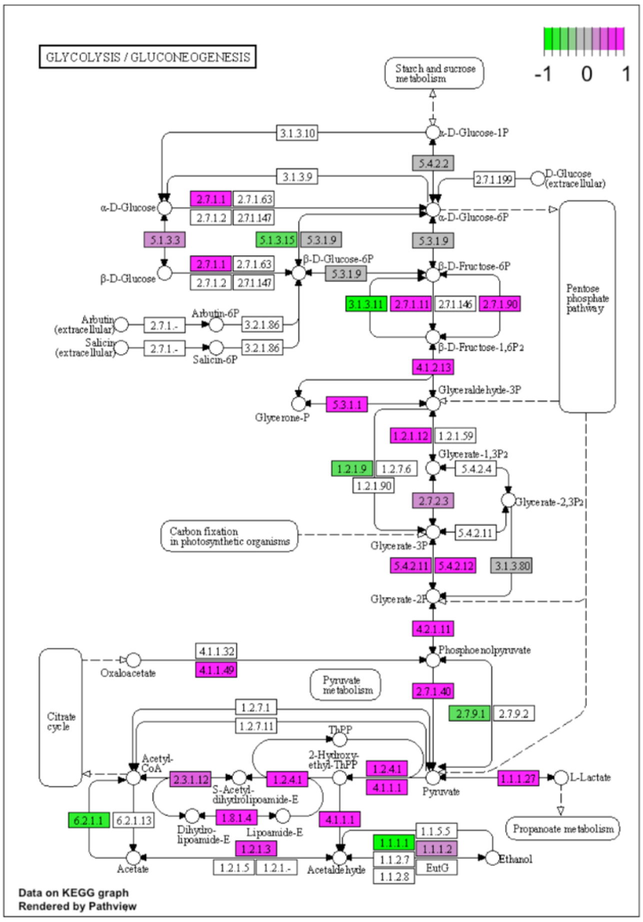

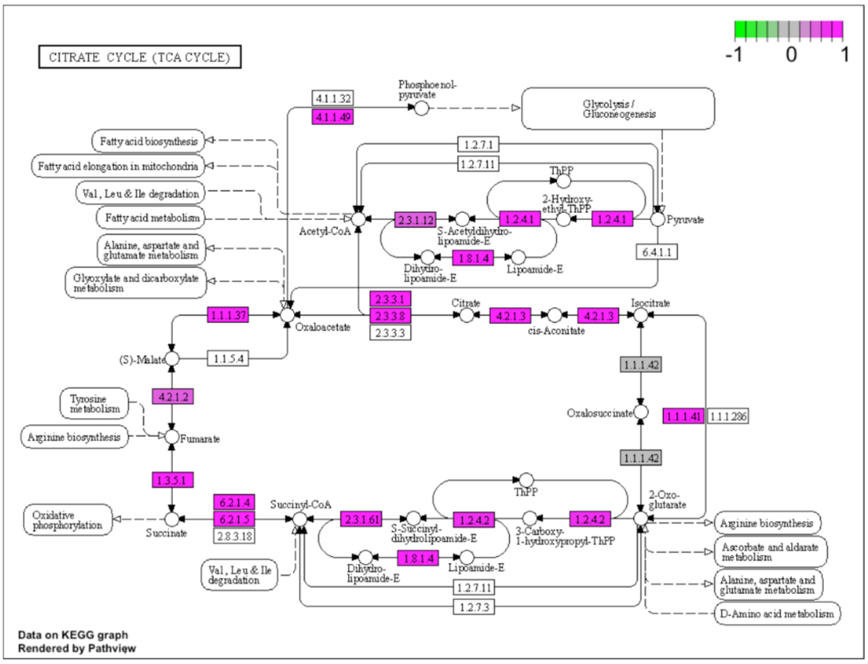

Supplemental Figure 5 (continued)

C ***rpoTmp***

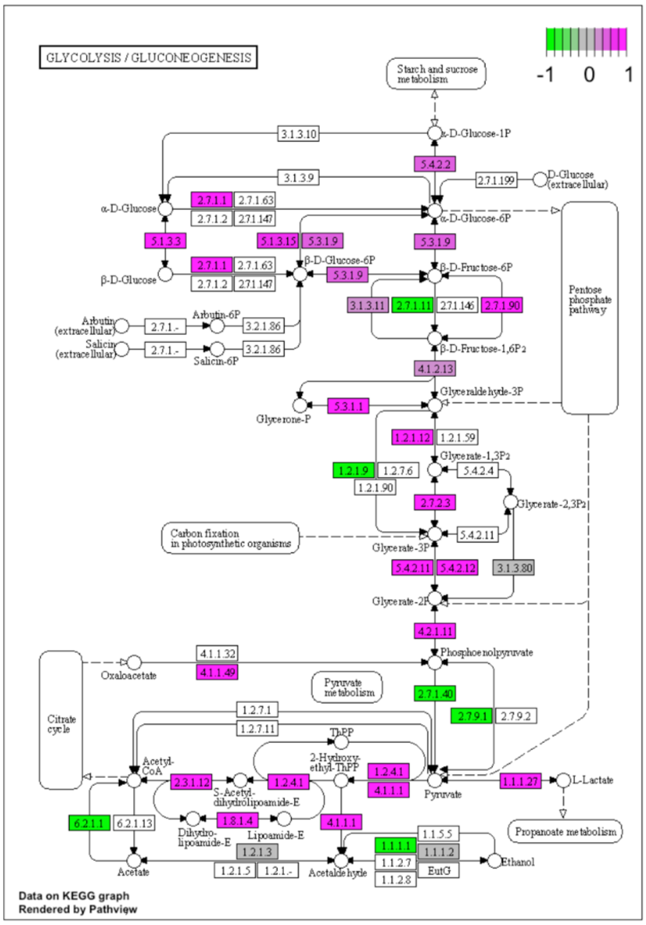

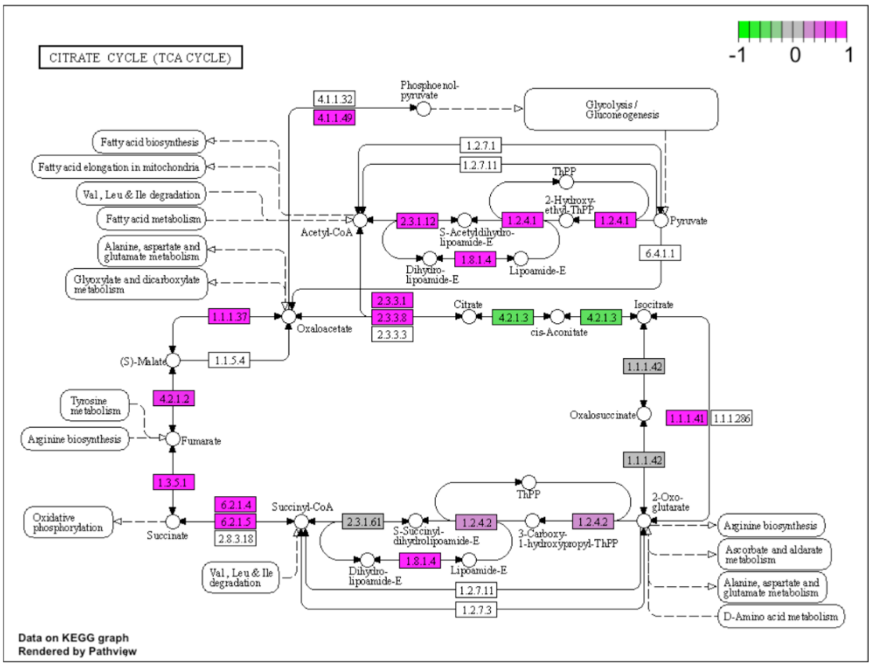

Supplemental Figure 5 (continued)

D ***rppr5***

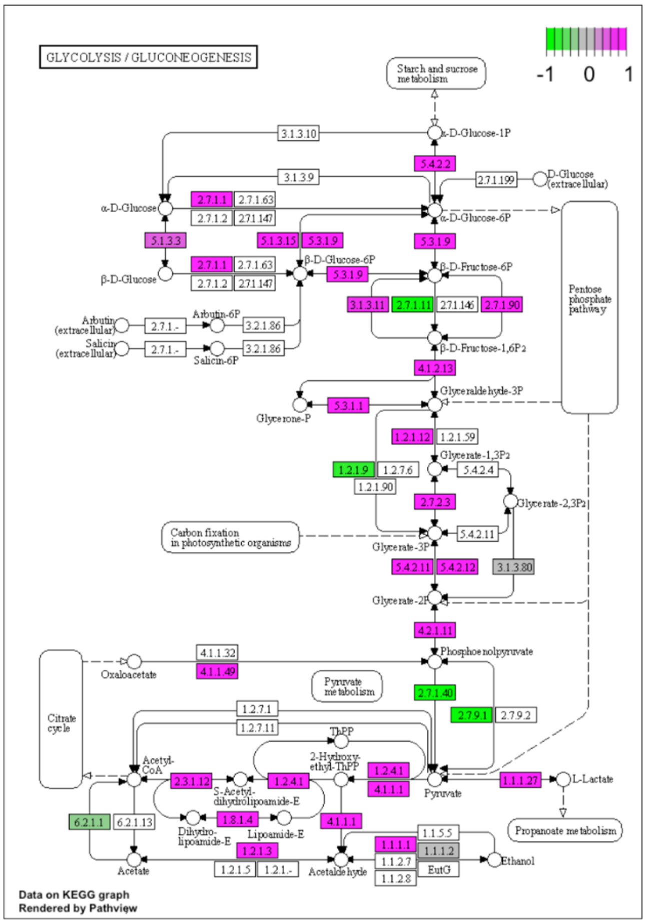

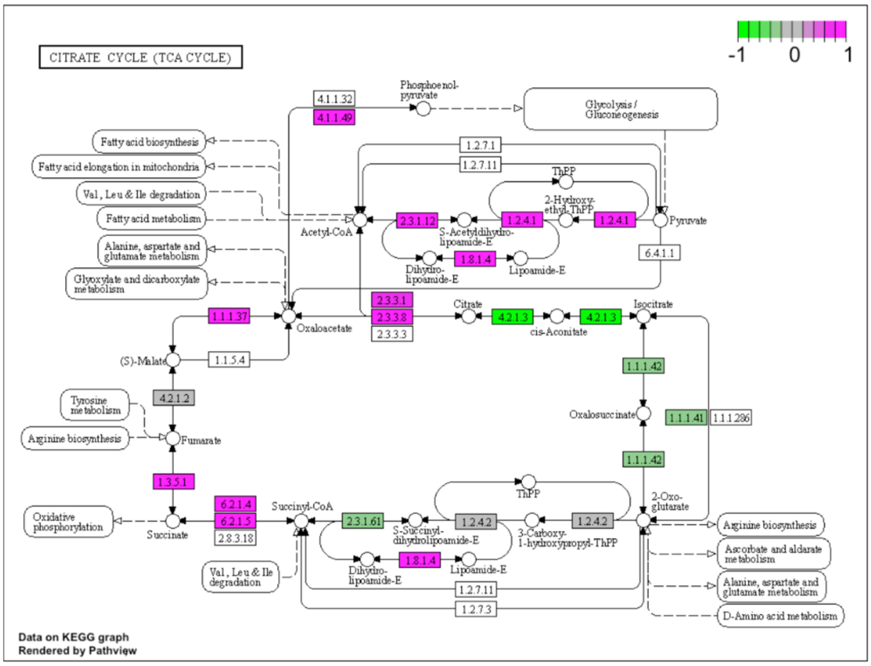

Supplemental Figure 5 (continued)

E ***wtf9***

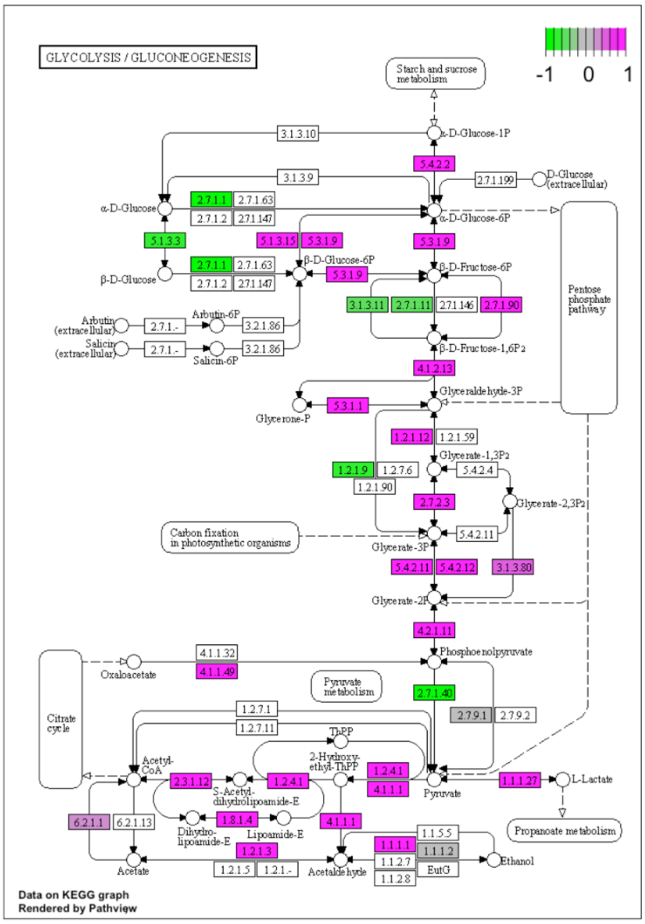

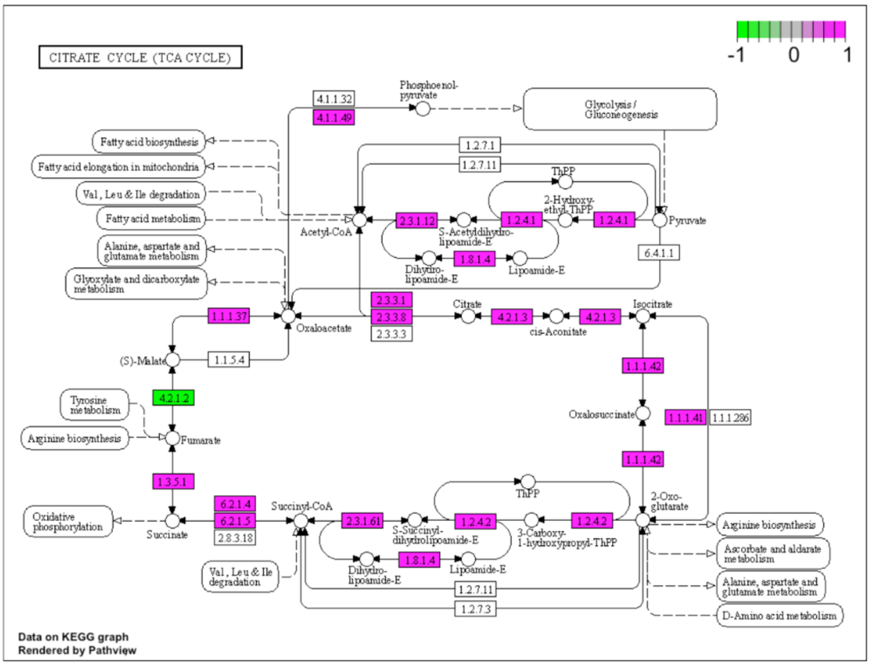

Supplemental Figure 5 (continued)

Supplemental Figure 5.

Pathway analysis of glycolysis (path: ath00010) and TCA cycle (path: ath00020)
A-E: Each cell corresponds to relative expression to WT. Red and Green indicate higher and lower expression to WT, calculated from log2 fold change of the transcript abundance.

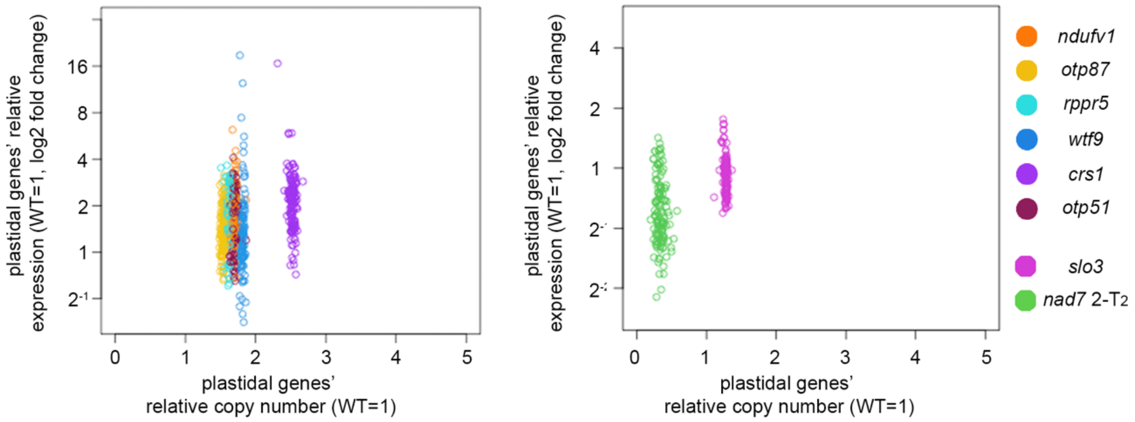

Supplemental Figure 6

Relationship between plastid genome copy number and expression. The horizontal axis is the relative copy number of organelle genes, and the vertical axis is the relative expression level of organelle genes. Each point on the scatter plot corresponds to a gene in the organelle genome.

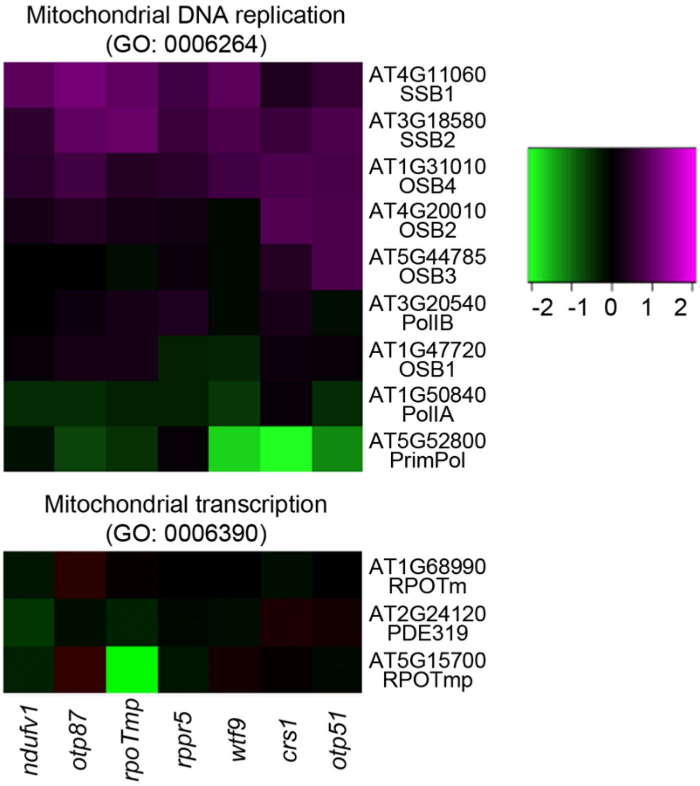

Supplemental Figure 7

Heatmaps of mitochondrial DNA replication and RNA transcription genes

Each cell corresponds to relative expression to WT. Red and Green indicate higher and lower expression to WT, calculated from difference in z-score. Genes with mitochondrial DNA replication term (GO: 0006264; upper panel), mitochondrial transcription term (GO: 0006390; lower panel) were shown.

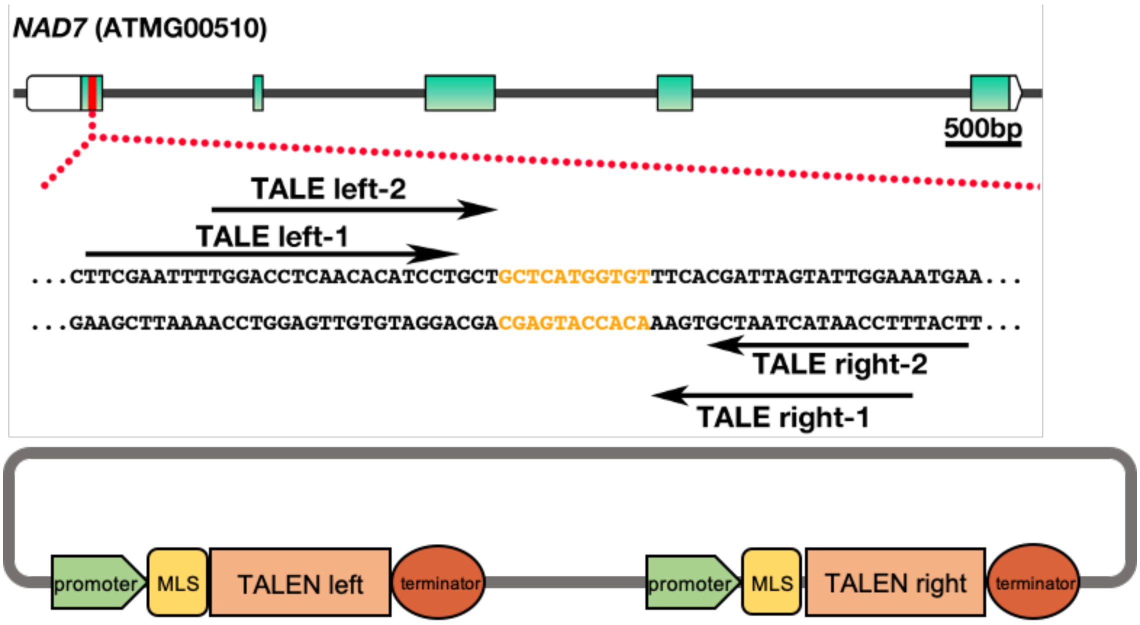

Vector construction

Vector No.	TALEN Left	TALEN Right	No. of T_1 plants	No. of T_1 plants transfected with each vector
#1	1	1	2	1- and 2-T_1
#2	1	2	13	3-, 4-, and 5-T_1
#3	2	1	7	None
#4	2	2	8	6- to 10-T_1

Supplemental Figure 8

The target sequence and schematic structure of mitoTALENs vectors in *NAD7* gene. Arrow shows the target sequence and yellow letters mean spacer sequence for TALEN. Table at the bottom indicates vector construction and combination of TALEN motifs.
